# Periplocymarin alleviates pathological cardiac hypertrophy via inhibiting the JAK2/STAT3 signalling pathway

**DOI:** 10.1111/jcmm.17267

**Published:** 2022-04-01

**Authors:** Cai‐lian Fan, Sui Liang, Meng‐nan Ye, Wan‐jun Cai, Miao Chen, Yun‐long Hou, Jun Guo, Yi Dai

**Affiliations:** ^1^ Department of Cardiology Jinan University First Affiliated Hospital Jinan University Guangzhou China; ^2^ 47885 College of Pharmacy Jinan University Guangzhou China; ^3^ National Key Laboratory of Collateral Disease Research and Innovative Chinese Medicine Shijiazhuang China

**Keywords:** JAK2/STAT3 signalling pathway, pathological cardiac hypertrophy, periplocymarin

## Abstract

Pathological cardiac hypertrophy is the most important risk factor for developing chronic heart failure. Therefore, the discovery of novel agents for treating pathological cardiac hypertrophy remains urgent. In the present study, we examined the therapeutic effect and mechanism of periplocymarin (PM)‐mediated protection against pathological cardiac hypertrophy using angiotensinII (AngII)‐stimulated cardiac hypertrophy in H9c2 cells and transverse aortic constriction (TAC)‐induced cardiac hypertrophy in mice. In vitro, PM treatment significantly reduced the surface area of H9c2 cells and expressions of hypertrophy‐related proteins. Meanwhile, PM markedly down‐regulated AngII‐induced translocation of *p*‐STAT3 into the nuclei and enhanced the phosphorylation levels of JAK2 and STAT3 proteins. The STAT3 specific inhibitor S3I‐201 or siRNA‐mediated depleted expression could alleviate AngII‐induced cardiac hypertrophy in H9c2 cells following PM treatment; however, PM failed to reduce the expressions of hypertrophy‐related proteins and phosphorylated STAT3 in STAT3‐overexpressing cells, indicating that PM protected against AngII‐induced cardiac hypertrophy by modulating STAT3 signalling. In vivo, PM reversed TAC‐induced cardiac hypertrophy, as determined by down‐regulating ratios of heart weight to body weight (HW/BW), heart weight to tibial length (HW/TL) and expressions of hypertrophy‐related proteins accompanied by the inhibition of the JAK2/STAT3 pathway. These results revealed that PM could effectively protect the cardiac structure and function in experimental models of pathological cardiac hypertrophy by inhibiting the JAK2/STAT3 signalling pathway. PM is expected to be a potential lead compound of the novel agents for treating pathological cardiac hypertrophy.

## 1 INTRODUCTION

Cardiac hypertrophy is a typical response of the cardiac muscle experiencing long‐term stress due to chronic pressure, volume overload or neuro‐hormonal stimulation.^1^ It can be categorized as physiological or pathological hypertrophy, both exhibiting markedly distinct outcomes. Physiological cardiac hypertrophy does not develop into heart failure, while pathological cardiac hypertrophy typically develops into heart failure and can even induce sudden death.^2^, ^3^ Accumulated evidence indicates that pathological cardiac hypertrophy invariably induces cardiomyocyte death, interstitial fibrosis, myocardial inflammation, diastolic and systolic dysfunction, accompanied by an increased size and mass of myocardial cells and up‐regulation of foetal gene (atrial natriuretic peptide [ANP], collagen I, β‐myosin heavy chain [β‐MHC]) expressions.^4^, ^5^, ^6^, ^7^ Therefore, exploring the underlying mechanism, and effective prevention and treatment measures for pathological myocardial hypertrophy, remains of considerable significance.

Numerous reports have confirmed that the renin‐angiotensin‐aldosterone system (RASS) plays a crucial role in the progression of cardiac hypertrophy.^8^, ^9^ AngiotensinII (AngII), a primary mediator of RASS, can induce an inflammatory phenotype in cardiomyocytes and lead to cellular hypertrophy, which could trigger the signal transduction of JAK2‐STAT3 pathway.^10^, ^11^, ^12^, ^13^, ^14^ The JAK2‐STAT3 signalling pathway participates in several biological processes induced by cytokines such as immune regulation, cell proliferation, differentiation and apoptosis.^15^, ^16^ Modern pharmacological studies have established that the JAK2/STAT3 signalling pathway participates in the progression of myocardial ischaemia, cardiac hypertrophy and heart failure.^17^, ^18^, ^19^ STAT3, a key transcription factor in this pathway, can be recruited and phosphorylated by JAK2, and the phosphorylated STAT3 protein can enter the nucleus as a dimer and bind with the target gene to regulate the transcription of downstream genes.^15^, ^20^ Recent research has revealed that cardiac hypertrophy can be effectively improved by inhibiting the activation of the JAK/STAT3 signalling pathway using STAT3 inhibitors or silencing the STAT3 gene.^14^, ^21^, ^22^ Therefore, STAT3 is regarded as a novel therapeutic target for delaying and reducing the progression from cardiac hypertrophy to chronic heart failure.

Natural products have shown a wide range of physiological activities, and are considered as the most important sources for new drug research and development.^23^ Periplocymarin (PM), a cardiac glycoside, is mainly derived from the traditional Chinese medicine Periplocae Cortex.^24^ Till date, the biological and pharmacological effects of PM have mostly focused on its anti‐cancer and cardiotonic activities.^24^, ^25^ A recent study has reported that PM can reduce isoproterenol (ISO)‐induced cardiac fibrosis and hypertrophy in C57BL/6 mice.^26^ The above‐listed findings suggest that PM may be a valuable and potential compound for treating cardiac hypertrophy. However, in‐depth studies on the therapeutic effects and underlying mechanisms of PM for ameliorating pathological cardiac hypertrophy are lacking.

Therefore, the therapeutic effect and associated molecular mechanisms of PM were examined using AngII‐induced in H9c2 cells and TAC‐induced cardiac hypertrophy in mice. Our results collectively demonstrated that PM prevented cardiac hypertrophy in vivo and in vitro by inhibiting the JAK2/STAT3 pathway.

## 2 MATERIALS AND METHODS

### 2.1 Materials

The primary antibodies targeting GAPDH, Collagen type I, α‐SMA and the second antibodies against rabbit and mouse lgG (H + L) were obtained from Servicebio. Antibodies targeting JAK2, *p*‐JAK2(Tyr1007/1008), STAT3, *p*‐STAT3(Tyr705), Histone H3 and the second Alexa Fluor 488‐labled antibody against rabbit were obtained from Beyotime Institute of Biotechnology. Monoclonal antibodies of TGF‐β1 and ANP were provided by Santa Cruz Biotechnology. PM was obtained from Chengdu Chroma‐Biotechnology Co., Ltd. (purity > 98%). AngII was obtained from Bide Pharmatech Ltd. Valsartan (Val) was provided by Jiangsu Aikon Biopharmaceutical R&D Co., Ltd. The selective STAT3 inhibitor, S3I‐201, was obtained from MedChem Express. Phalloidin‐iFluor 488 Reagent and DAPI were obtained from Fangyuan Biotechnology Co., Ltd.

### 2.2 Cell culture and treatment

Guangzhou Cellcook Biotech Co., Ltd. provided H9c2 rat heart‐derived cells. H9c2 cells were carefully cultured in a stable environment with DMEM added with 10% FBS and 1% penicillin‐streptomycin and kept in an incubator at 37℃ and 5% CO_2_. Before cells were addressed by PM, DMEM containing 3% FBS replaced the original normal medium. Subsequently, 1 μM AngII was added into the culture medium for 24 h.^27^, ^28^

### 2.3 MTT assay

MTT method was used to assess cellular viability in this study. Briefly, a density of 5000 H9c2 cells/well was seeded in 96‐well plates. Then, H9c2 cells were attached for 24 h before being treated by PM at concentrations of 12.5, 25, 50, 100 μM for 24 h. After that, 20 μl MTT was directly added into each well and the plates were kept culturing for 4 h. The medium was removed, and 150 μl DMSO was added. Finally, the OD values of cells were determined at 490 nm with the help of a microplate reader (BioTeck).

### 2.4 Western blot analysis

The left ventricular tissues of mice or H9c2 cells were lysed on ice using RIPA lysis buffer containing with 1mM phenylmethylsulfonyl fluoride (PMSF) and phosphate inhibitor. Cytoplasmic and nuclear proteins of H9c2 cells were separated using a protein extraction kit (BestBio). After treatment with PM, left ventricular tissues of mice or H9c2 cells were collected and digested using RIPA solution. Samples were then quickly centrifuged at 14,000 rpm for 10 min, and total proteins were harvested. The total protein concentrations were quantified using the BCA Protein Assay kit (Beyotime). Equal amounts of protein samples (25 μg of cellular protein and 50 μg of tissue protein) from different groups were used to do Western blot experiments as follows: Proteins were separated using 10% or 12% sodium dodecyl sulphate‐polyacrylamide gel electrophoresis (SDS‐PAGE) gels for electrophoresis, and then quickly transferred to polyvinylidene fluoride (PVDF) membranes. Subsequently, 1× tris‐buffered saline with 1% Tween‐20 (TBST) and 5% de‐fatted milk powder was used to block membranes. The membranes were then washed with TBST buffer, followed by incubation with specific primary antibodies at 4℃ for 12 h. Next, membranes were washed again with TBST buffer and incubated with the secondary antibodies for 2 h on a shaker. Finally, membranes were washed with TBST prior to subsequent exposure. The protein bands were exposed to chemiluminescence developing agents and quantified using the ImageJ software (National Institutes of Health). GAPDH and histone H3 were used as internal controls.

### 2.5 Phalloidin staining

Phalloidin staining was used to observe morphological changes in H9c2 cells. In brief, H9c2 cells at a density of 2 × 10^4^ cells/well were cultured in 6‐well plates and treated with 1 μM AngII for 24 h. Subsequently, 4% paraformaldehyde was used to fix cells for 30 min on ice. Then, PBS containing 0.1% Triton X‐100 was added for 10 min to permeabilize the cells. Next, the cells were stained with phalloidin staining solution (5 μg/ml) for 30 min. In addition, nuclei were stained with DAPI at room temperature in the dark for 10 min. The cells were then quickly washed with PBS three times, and morphological changes in H9c2 cells were captured using a fluorescence microscope.

### 2.6 STAT3 overexpression and STAT3 shRNA plasmids transfection

STAT3‐overexpressing pcDNA3.1 and STAT3 shRNA plasmids were obtained from Hunan Fenghui Biology Co., Ltd. Detailed information of plasmids was provided in Figure S1. H9c2 cells were seeded at a density of 3 × 10^5^cells/well in 6‐well plates. Next, the medium containing 10% FBS was removed, and Opti‐MEM I medium (Gibco) was added into the wells when cell confluency exceeded 90%. STAT3 overexpression and STAT3 shRNA plasmids were transfected into H9c2 cells using Lipofectamine 2000 (1: 2.5, Invitrogen) according to the manufacturer's protocol. Subsequently, Opti‐MEM I reduced‐serum medium was replaced with normal medium after 6 h. Cell lysates from different groups were analysed by immunoblotting with GAPDH as a reference to assess STAT3 overexpression and STAT3 siRNA efficiency.

### 2.7 Animals’ experiments

Male C57BL/6N mice (22–25 g, 6–8 weeks) were selected from Beijing Vital River Laboratory Animal Technology Co. Ltd. All animals were housed and fed by Animal Center of Shijiazhuang YiLing Pharmaceutical Co., Ltd. All these experiments about animals were in accord with the guidance of Guide for the Care and Use of Laboratory Animals. In addition, the project about animals was approved by the Committee on the Ethics of Animal Experiments of Shijiazhuang YiLing Pharmaceutical Co., Ltd under approval reference number No. N2020213.

All mice were kept in a clean room with stable temperature and 12 h light/dark cycle. After adapted to the environment for a week, the mice were randomized into four groups (*n* = 10): a. sham‐operated group; b. transverse aortic constriction (TAC) model group; c. PM (10 mg/kg/day) group; d. S3I‐201 (5 mg/kg/day) group. The sham‐operated animals underwent the same operation as the TAC group animals, except that the aortas were not ligated in the last step. Seven days after surgery, the mice were given intragastric administration consecutively for 21 days in the PM group and S3I‐201 group. After administration for 3 weeks, all mice were sacrificed and heart tissues were taken immediately. Likewise, the tibias were removed and length were recorded for calculation of HW/BW and HW/TL. Then, one part of the left ventricular tissues was immersed into 10% formalin for analysis of histological changes, the other part of the left ventricular tissues was quickly frozen using liquid nitrogen for western blot analysis.

### 2.8 Histological analysis

Haematoxylin‐eosin (HE) and Masson staining were performed to analyse changes in the cardiac structure. The left ventricular tissues of mice were harvested by removing the right ventricular and atria. Then, samples were soaked in 10% formalin for 48 h and embedded in paraffin. Tissues were cut into 4‐μm‐thick sections and stained with HE for morphological observations. Masson trichrome staining was performed to estimate fibrosis as described previously.^21^ All changes in heart tissues were observed and captured using an Olympus BH2 microscope (Olympus Optical Co. Ltd.).

### 2.9 Wheat germ agglutinin staining

The left ventricular tissues of mice were obtained and quickly soaked in 10% formalin for 48 h. After embedding in paraffin, the tissues were cut into 4‐μm‐thick sections. Sections were then dewaxed in xylene, hydrated through a graded series of ethanol to water and incubated for 30 min with wheat germ agglutinin (WGA) staining (5 μg/ml) performed in the dark. After washing the slides three times with PBS, DAPI was used to stain cell nuclei. The slides were then observed under a fluorescence microscope (Olympus Optical Co. Ltd.).

### 2.10 Statistical analysis

Data were analysed using one‐way analysis of variance (ANOVA), followed by Tukey's post hoc test using GraphPad 5.0 software (GraphPad Software, Inc.). Data values are presented as mean ± standard deviation (SD). Statistical significance was set at *p* < 0.05.

## 3 RESULTS

### 3.1 PM played a protective role in AngII‐induced hypertrophy of H9c2 cells

**FIGURE 1:**
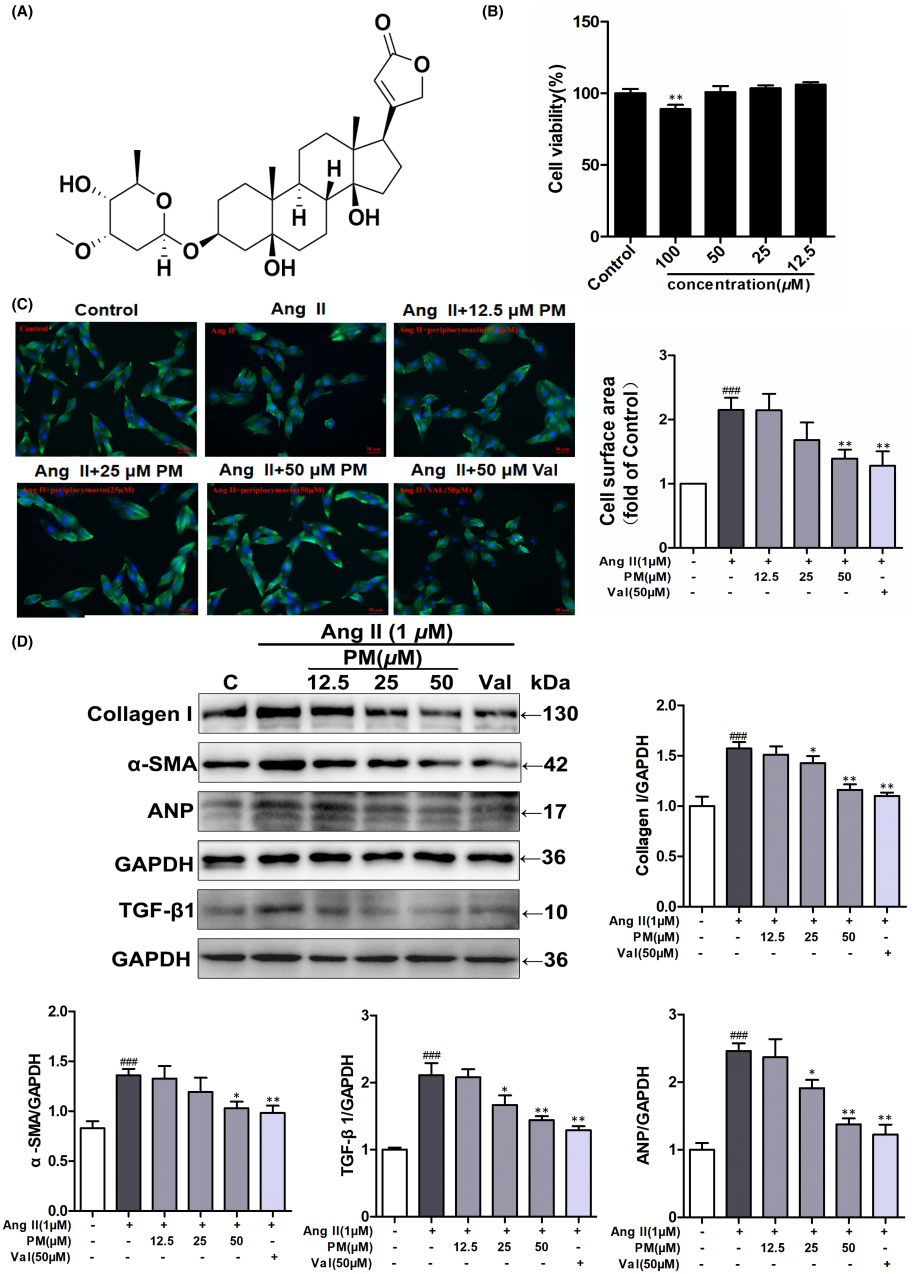
Periplocymarin (PM) attenuated AngⅡ‐induced hypertrophic responses of H9c2 cells. (A) The chemical structure of PM. (B) H9c2 cells were treated with different concentrations of PM for 24 h. Cell viability was measured by MTT assay. (C) H9c2 cells were pretreated with PM (12.5, 25, 50 μM) and 50 μM Val for 3 h, cells were then exposed to 1 μM AngⅡ for 24 h. Cell surface area was measured by phalloidin staining. Bars: 50 μm. (D) The expressions of collagen I, α‐SMA, TGF‐β1 and ANP were detected by Western blot. ^#^ *p* < 0.05, ^##^ *p* < 0.01 and ^###^ *p* < 0.001 vs. control group. *p < 0.05, **p < 0.01 vs. AngⅡ group. Data were presented as the mean ± SD, *n* = 3

The cytotoxic effect of PM (Figure 1A) was assessed using the MTT assay in H9c2 cells exposed to different PM concentrations (12.5, 25, 50 and 100 μM) for 24 h, measuring the relative cell viability. The results showed that treatment with 100 μM PM significantly inhibited cell viability (*p* < 0.01), while other examined concentrations did not demonstrate any toxic effect (Figure 1B). Thus, concentrations of 12.5, 25 and 50 μM PM were used to assess its effects on cardiac hypertrophy. In the present study, valsartan (Val) was used as a positive control drug. H9c2 cells were pre‐treated with different concentrations of PM (12.5, 25 and 50 μM) and Val (50 μM) for 3 h, followed by treatment with 1 μM AngII for 24 h. Notably, AngII treatment increased the cardiomyocyte surface area, as well as elevated protein levels of Collagen I, α‐SMA, TGF‐β1 and ANP; however, pre‐treated with 25 and 50 μM PM and Val markedly suppressed the enlargement of AngII‐induced cell surface area and expressions of these hypertrophic proteins (Figure 1C,D). Collectively, these findings indicated that PM could protect against AngII‐induced cardiac hypertrophy in a concentration‐dependent manner.

### 3.2 PM suppressed AngII‐induced JAK2 and STAT3 phosphorylation

**FIGURE 2:**
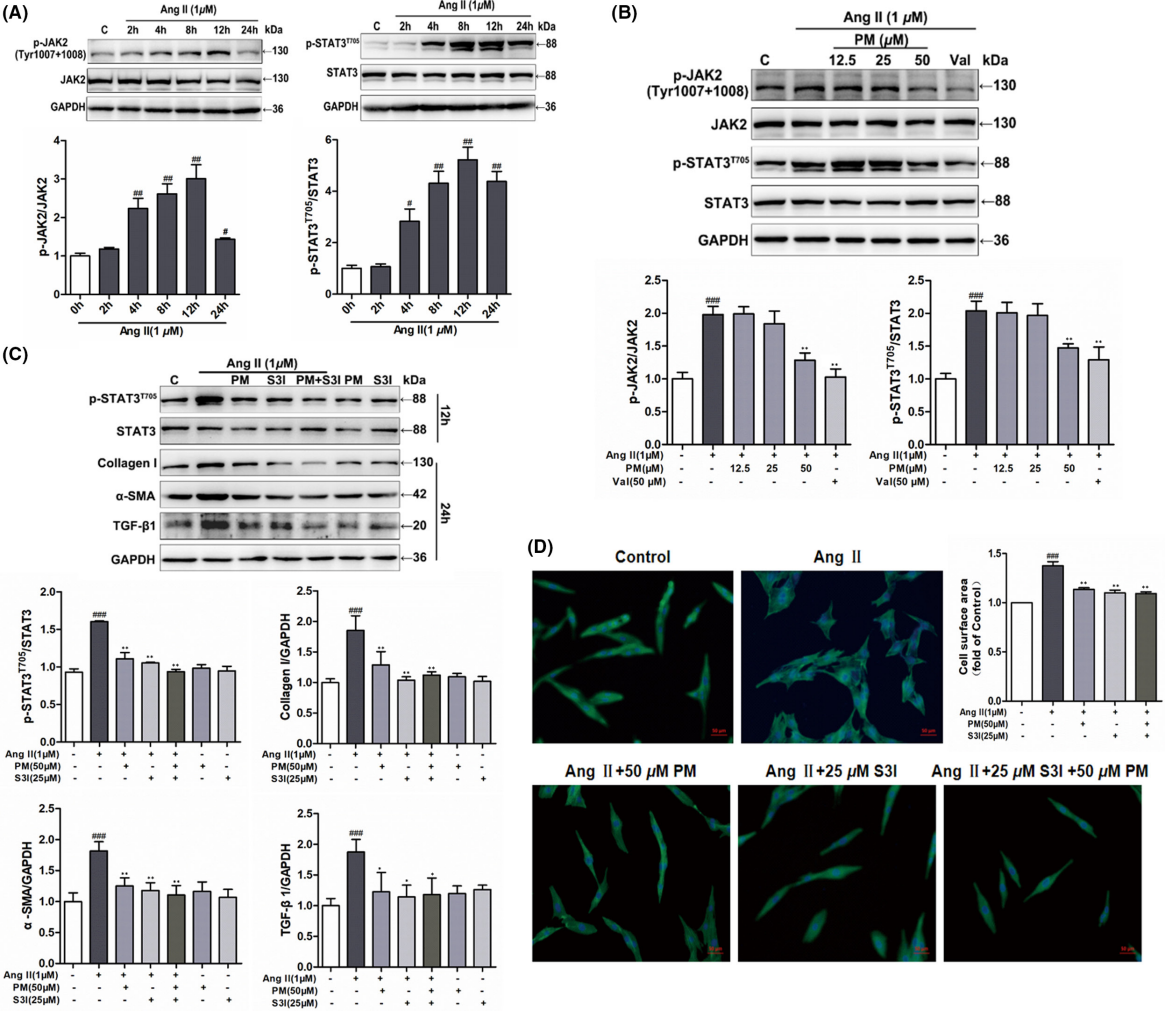
Periplocymarin (PM) suppressed AngⅡ‐induced phosphorylation of JAK2 and STAT3. (A) H9c2 cells were incubated with 1 μM AngⅡ for 2, 4, 8, 12 and 24 h respectively. The phosphorylation of JAK2 (Tyr1007 + 1008) and STAT3 (Tyr705) was detected by Western blot. (B) H9c2 cells were pretreated with PM (12.5, 25, 50 μM) and 50 μM Val for 3 h followed by 1 μM AngⅡ for 12 h. The expressions of *p*‐JAK2 (Tyr1007 + 1008) and *p*‐STAT3 (Tyr705) were measured by Western blot. (C) H9c2 cells were treated with 1 μM AngⅡ in the presence of 50 μM PM, 25 μM S3I and 50 μM PM + 25 μM S3I. The protein levels of *p*‐STAT3 (Tyr705) and hypertrophic markers were measured. (D) Assessment of cell area changes through phalloidin staining. Bars: 50 μm. ^#^ *p* < 0.05, ^##^ *p* < 0.01 and ^###^ *p* < 0.001 vs. control group. *p < 0.05, **p < 0.01 vs. AngⅡ group. Data were presented as the mean ± SD, *n* = 3

Numerous studies have shown that the JAK2/STAT3 signalling pathway is a key signal transduction pathway known to participate in the progression of cardiac hypertrophy.^19^, ^22^ STAT3 activation was shown to have an important effect on the regulation of hypertrophic growth.^14^ Therefore, inhibiting the activation of STAT3 is considered a potentially effective strategy for alleviating cardiac hypertrophy. Previously, we have found that PM could inhibit IL‐6‐induced STAT3 expression in HepG 2 cells with an IC_50_ value of 49.3 μM (Figure S2), and PM had a good binding affinity with STAT3 by molecular virtual docking analysis (Figure S3). Therefore, we speculated that PM improved cardiac hypertrophy possibly by inhibiting STAT3 activation. Then, the phosphorylation of STAT3(Tyr705) and JAK2(Tyr1007/1008) were examined to elucidate the mechanism which PM alleviates cardiac hypertrophy. In H9c2 cells, AngII increased the expressions of *p*‐JAK2(Tyr1007/1008) and *p*‐STAT3 (Tyr705) at 4 h, and both reached maximum levels at 12 h (Figure 2A). Treatment with 50 μM PM led to a significant decrease in *p*‐JAK2(Tyr1007/1008) and *p*‐STAT3 (Tyr705) levels, whereas PM did not reduce the total protein expressions of JAK2 and STAT3 (Figure 2B). To further explore our hypothesis, S3I‐201, a selective STAT3 inhibitor,^14^ was used as a positive drug to determine whether a STAT3 inhibitor could mimic the anti‐hypertrophic effect of PM. As shown in Figure 2C, S3I‐201 exerted similar effects to PM in terms of reducing AngII‐induced JAK2 and STAT3 phosphorylation and cell surface area. These observations suggested that PM played the role in alleviating cardiac hypertrophy, which was related to the inhibition of the JAK2/STAT3 signalling pathway.

### 3.3 PM inhibited nuclear translocation of STAT3 in H9c2 cells

**FIGURE 3:**
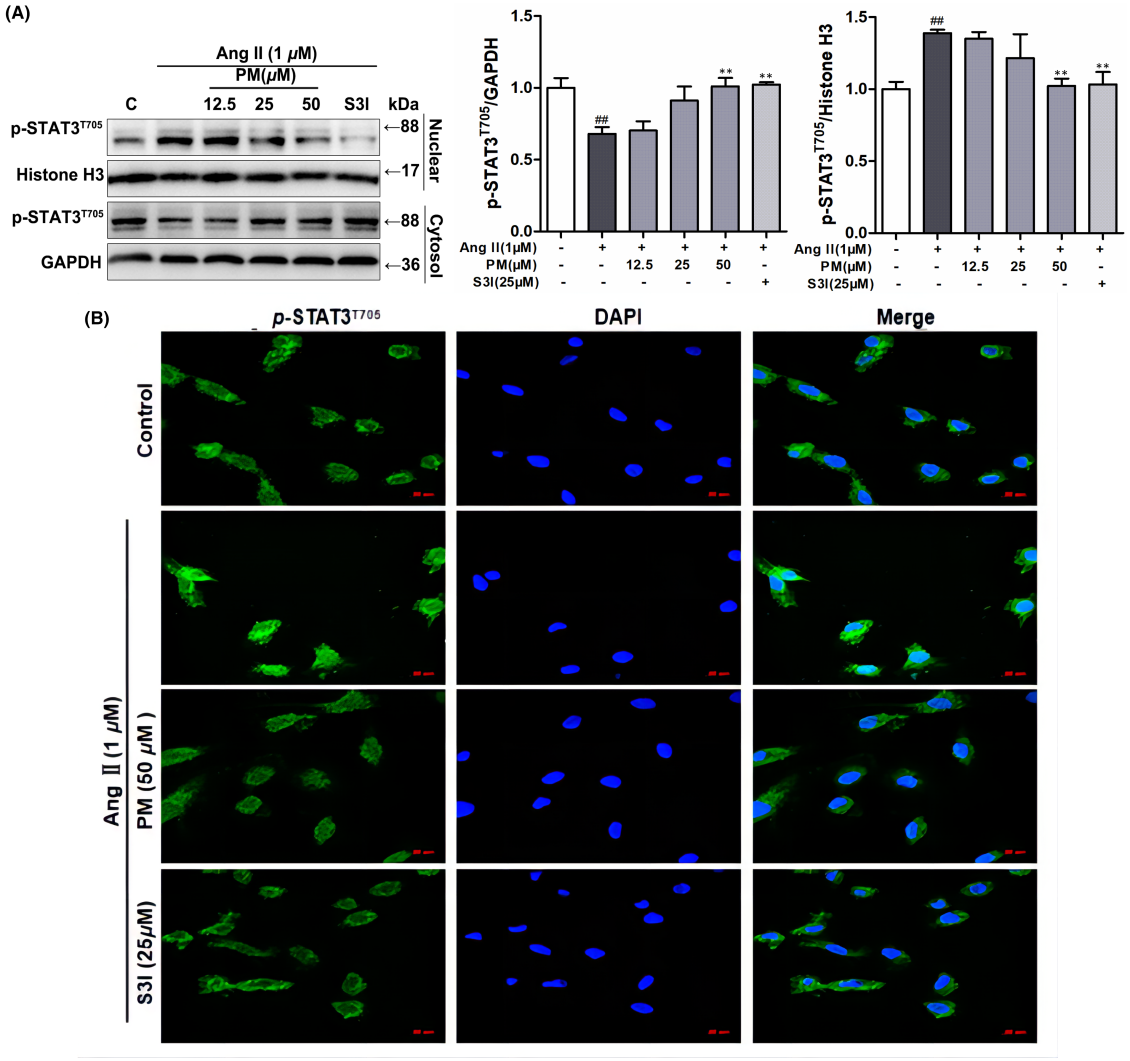
Periplocymarin (PM) inhibited AngⅡ‐induced nuclear translocation of STAT3. (A) H9c2 cells were preincubated with PM (12.5, 25, 50 μM) and 25 μM S3I for 3 h, then exposed to 1 μM AngⅡ for 12 h. The expression of *p*‐STAT3 (Tyr705) in nuclear fraction and cytoplasmic fraction was detected by Western blot. (B) The subcellular localization of STAT3 was visualized by immunofluorescence assay. Bars: 50 μm. ^#^ *p* < 0.05, ^##^ *p* < 0.01, ^###^ *p* < 0.001 vs. control group. *p < 0.05, **p < 0.01 vs. AngⅡ group. Data were presented as the mean ± SD, *n* = 3

It has been reported that once STAT3 is activated via phosphorylation, STAT3 forms dimers to interact with the reciprocal phosphotyrosine‐SH2 domain, which quickly translocate to the nucleus, where STAT3 binds to DNA and induces the expression of target genes.^15^, ^20^ Hence, STAT3 distribution in the nuclear and cytosolic fractions was analysed. As shown in Figure 3A, PM effectively decreased AngII‐mediated nuclear localization of STAT3. AngII reduced the cytosolic levels of STAT3 and PM maintained this activity. Moreover, the effect of PM on nuclear STAT3 translocation was observed using immunofluorescence staining. Likewise, 50 μM PM successfully suppressed AngII‐induced nuclear translocation of *p*‐STAT3 in H9c2 cells (Figure 3B). These results further indicated that STAT3 signalling was involved in the progression of AngII‐induced cardiac hypertrophy, and PM reduced AngII‐induced cardiac hypertrophy by suppressing STAT3 activity, as determined from phosphorylation and nuclear translocation levels.

### 3.4 PM engages STAT3 in inhibiting AngII‐induced cardiomyocyte remodelling

**FIGURE 4:**
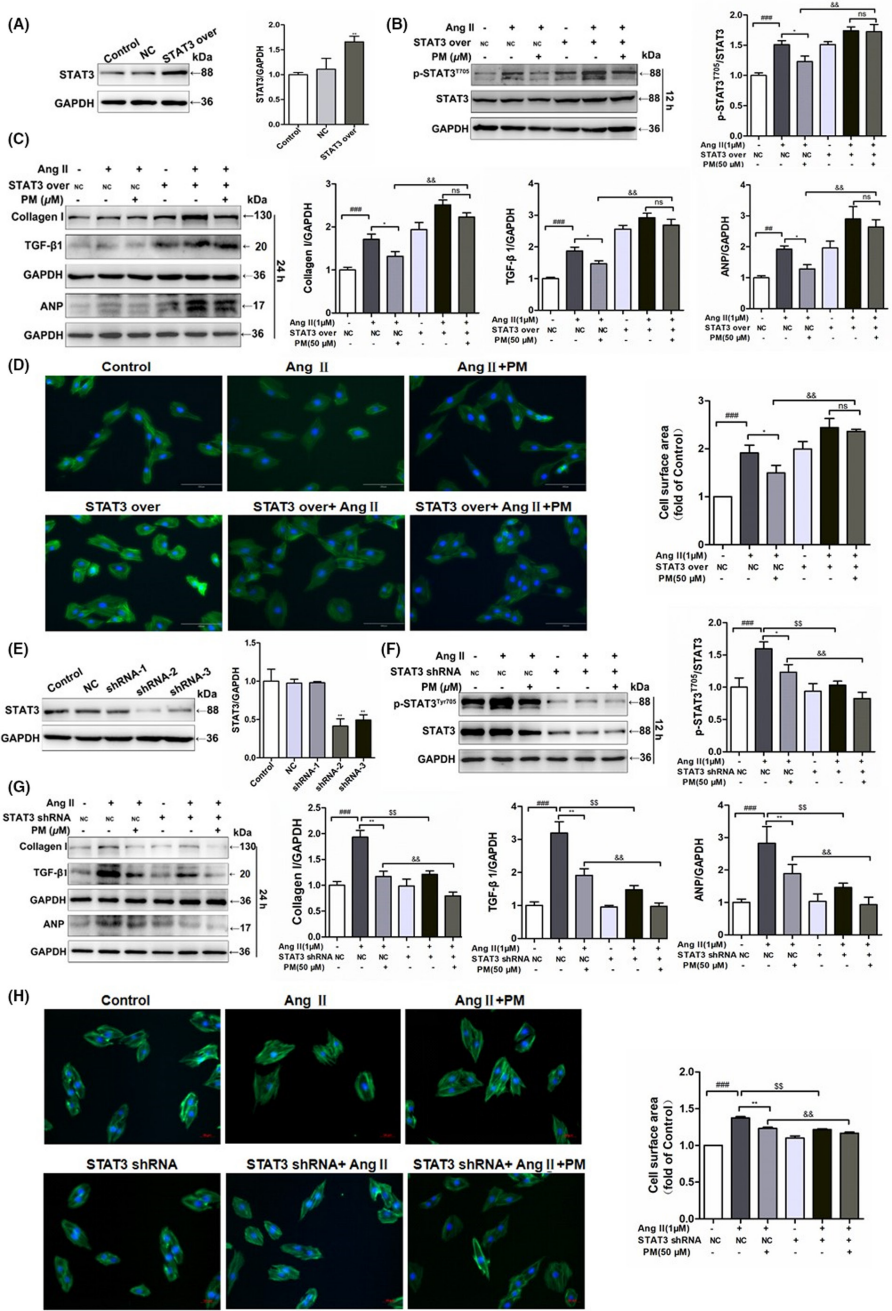
Periplocymarin (PM) engaged STAT3 in inhibiting AngⅡ‐induced cardiomyocyte remodelling. (A) H9c2 cells were transfected STAT3 overexpression plasmids and control cells were transfected with empty vector. Western blot was used to assess STAT3 expression. (B) H9c2 cells transfected STAT3 over‐expression plasmids and negative control were treated with PM for 3 h and then submitted to 1 μM AngⅡ for 12 h or 24 h. The expression of *p*‐STAT3 (Tyr705) was measured by Western blot. (C) Immunoblot analysis of Collagen I, TGF‐β1 and ANP following STAT3 over‐expression. (D) Representative phalloidin staining for cell surface area. Bars: 50 μm. (E) Western blot analysis of H9c2 cells which were submitted to different sequence of shRNA against STAT3. (F) H9c2 cells transfected STAT3 shRNA‐2 plasmids and empty plasmid were treated with PM for 3 h and then submitted to 1 μM AngⅡ for 12 h or 24 h. Western blot analysis for *p*‐STAT3 (Tyr705) level. (G) Effects of STAT3 shRNA on Collagen I, TGF‐β1 and ANP protein levels. (H) Phalloidin staining of STAT3 knockdown H9c2 cells. ^#^ *p* < 0.05, ^##^ *p* < 0.01, ^###^ *p* < 0.001 vs. NC group; *p < 0.05, **p < 0.01, ^$^ *p* < 0.05, ^$$^ *p* < 0.01 vs. NC + AngⅡ group; ^&^ *p* < 0.05, ^&&^ *p* < 0.01 vs. NC + AngⅡ + PM group. Data were presented as the mean ± SD, *n* = 3

To further elucidate the key role of STAT3 in cardiac hypertrophy, STAT3 overexpression and STAT3 shRNA plasmids were used to transfect H9c2 cells. Following the successful transfection of the STAT3 overexpression plasmids into H9c2 cells, the expression of STAT3 was notably up‐regulated (Figure 4A). Meanwhile, overexpression of STAT3 in H9c2 cells alone induced similar phenomena to AngII‐mediated changes in cell surface area and related protein expressions, aggravating AngII‐induced expansion of the cellular surface area and protein levels of *p*‐STAT3 and hypertrophic markers (Collagen I, TGF‐β1 and ANP); however, PM failed to reduce the levels of TGF‐β1, Collagen I, ANP, phosphorylated STAT3 proteins and cellular surface area in STAT3‐overexpressing cells (Figure 4B–D). In contrast, STAT3 shRNA‐2 exhibited the strongest STAT3 inhibition among the three STAT3 shRNA plasmids (Figure 4E), significantly reducing the increase in cell surface area and expression levels of *p*‐STAT3 and hypertrophic proteins induced by AngII, presenting a similar effect to PM (Figure 4F–H). Collectively, these findings suggested that PM improved AngII‐mediated cardiac hypertrophy by modulating STAT3.

### 3.5 PM protected against TAC‐induced cardiac hypertrophy and fibrosis in male C57BL/6N mice

**FIGURE 5:**
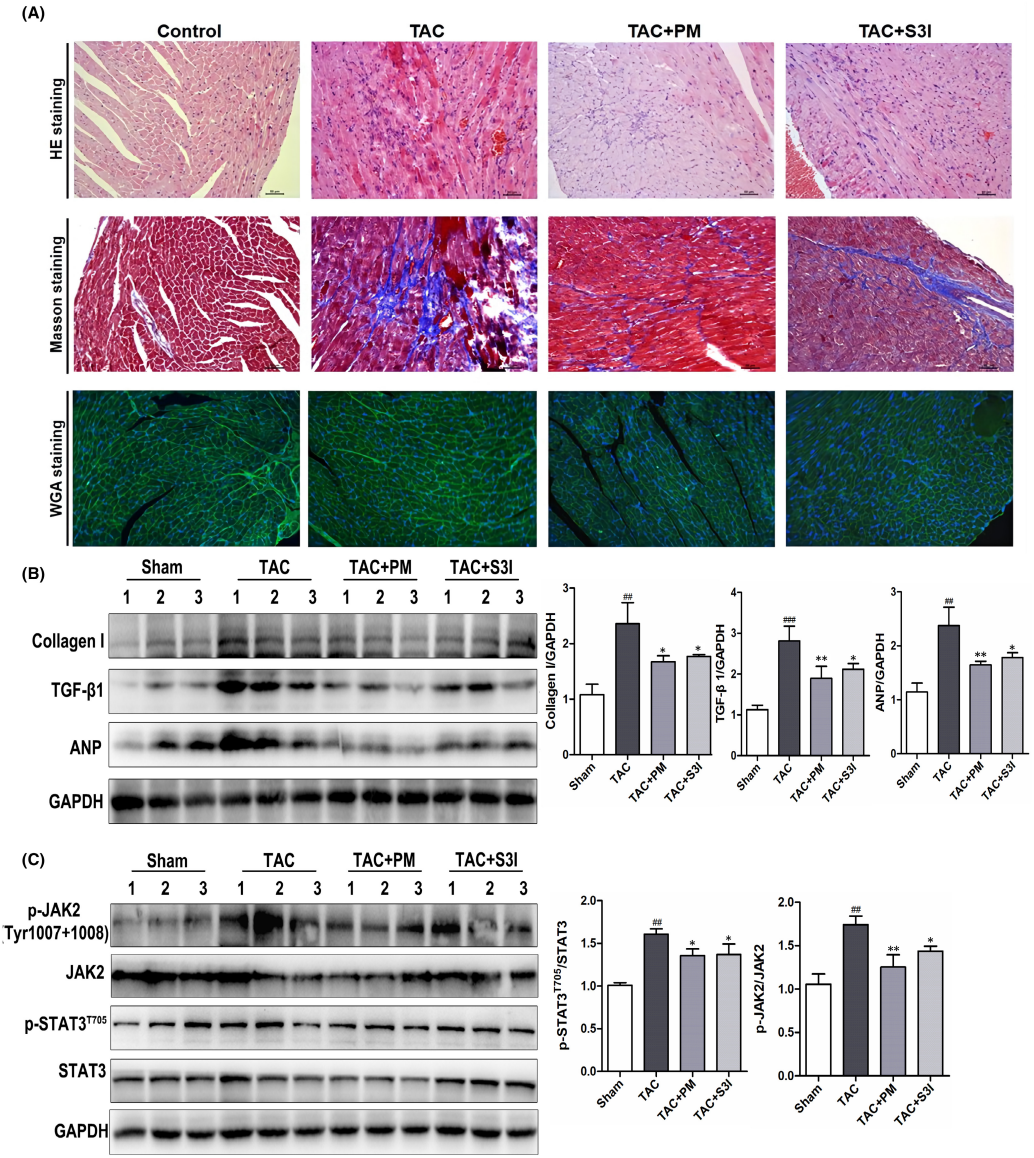
Periplocymarin (PM) protected against transverse aortic constriction (TAC)‐induced cardiac hypertrophy and fibrosis in mice (A) Representative micrographs of HE, Masson and WGA staining were shown (20×). Bars: 50 μm. (B) Western blotting analysis of Collagen I, TGF‐β1 and ANP proteins in heart tissues. (C) The protein levels of *p*‐JAK2, JAK2, *p*‐STAT3 and STAT3 were measured by Western blot. *n* = 3. ^#^ *p* < 0.05, ^##^ *p* < 0.01, ^###^ *p* < 0.001 vs. sham group. *p < 0.05, **p < 0.01 vs. TAC group. Data were presented as the mean ± SD

In order to further clarify the therapeutic effect and mechanism of PM in pathological cardiac hypertrophy, TAC‐induced cardiac hypertrophy was established in mice. The STAT3 inhibitor, S3I‐201, was used as a positive control to determine whether STAT3 inhibition could mimic PM activity. During the experimental period, the mortality rate of TAC‐induced animals was 16.7%. Cardiac function was assessed using echocardiography. TAC caused a significant decrease in left ventricular ejection fraction (LVEF), fractional shortening (FS), left ventricular end‐systolic posterior wall thickness (LVAWs) and left ventricular end‐diastolic anterior wall thickness (LVAWd), while both PM and S3I‐201 greatly improved these changes (Table 1). In addition, left ventricular end‐systolic volume (LVESV) and left ventricular end‐diastolic volume (LVEDV) were significantly increased (*p* < 0.01) in the TAC group when compared with the sham‐operated group; PM treatment significantly decreased LVESV and LVEDV (*p* < 0.05). These results suggested that PM can improve cardiac function in TAC mice. Furthermore, TAC‐induced mice displayed a significant increase in heart mass, while the ratios of heart weight to body weight (HW/BW) and heart weight to tibial length (HW/TL) were reduced following treatment with PM (Table 1). Although S3I‐201 reduced these indices, significant differences were not observed in the present study (Table 1). HE, Masson and WGA staining revealed that the absence of fibrosis and cardiac hypertrophy occurred in the sham group, while marked interstitial fibrosis and cardiac hypertrophy were detected in TAC‐treated mice; PM ameliorated these histopathological alterations (Figure 5A). Furthermore, results from Western blotting showed that PM and S3I‐201 reduced TAC‐induced hypertrophic and fibrosis markers (ANP, Collagen I, TGF‐β1) in mouse hearts (Figure 5B). As expected, TAC surgery also up‐regulated JAK2 and STAT3 phosphorylation, whereas these proteins were down‐regulated following PM and S3I‐201 administration (Figure 5C). Above results demonstrated that PM prevented TAC‐induced cardiac remodelling by inhibiting the JAK2/STAT3 signalling pathway.

**TABLE 1:** Effects of PM on changes of cardiac function in TAC‐induced mice

	Control	TAC		
			PM (10 mg/kg/day)	S3I (5 mg/kg/day)
	*n* = 10	*n* = 7	*n* = 9	*n* = 8
Heart rate, bpm	439.23 ± 37.15	418.33 ± 12.91	427.09 ± 16.81	418.09 ± 18.31
EF, %	74.53 ± 5.60	33.28 ± 2.63^##^	58.82 ± 8.74**	47.97 ± 2.61
FS, %	50.81 ± 2.69	18.22 ± 3.20^##^	38.83 ± 2.19**	26.43 ± 2.48
LVESV, μl	21.50 ± 6.41	35.82 ± 5.53^##^	28.36 ± 4.96^#^	30.91 ± 5.42
LVEDV, μl	73.42 ± 5.70	95.80 ± 9.02^#^	82.51 ± 7.08^#^	89.71 ± 9.31
LVAWs, mm	1.74 ± 0.18	0.98 ± 0.16^##^	1.43 ± 0.09*	1.35 ± 0.04*
LVAWd, mm	1.43 ± 0.07	0.77 ± 0.05^##^	1.21 ± 0.06**	1.00 ± 0.07*
HW/BW, mg/g	4.76 ± 0.18	9.91 ± 2.58^#^	7.73 ± 0.53*	8.85 ± 1.57
HW/TL, mg/mm	7.06 ± 0.29	14.19 ± 1.90^##^	11.04 ± 0.87*	11.96 ± 1.78

Note

The cardiac function was assessed using echocardiography in mice, and the parameters of heart rate, LVESV, LVEDV, LVEF, FS, LVAWs, LVAWd, HW/BW and HW/TL were recorded. ^#^ *p* < 0.05, ^##^ *p* < 0.01 vs. Control group. *p < 0.05, **p < 0.01 vs. TAC group. Data were presented as mean ± SD (*n* = 7 ~ 10).

Abbreviations: EF, ejection fraction; FS, fractional shortening; HW/BW, heart weight to body weight; HW/TL, heart weight to tibial length; LVAWd, left ventricular end‐diastolic anterior wall thickness; LVAWs, left ventricular end‐systolic posterior wall thickness; LVEDV, left ventricular end‐diastolic volume; LVESV, left ventricular end‐systolic volume; PM, periplocymarin; TAC, transverse aortic constriction.

## 4 DISCUSSION

Periplocymarin, a cardiac glycoside from Periplocae Cortex, has shown potentially beneficial effects for improving cardiovascular diseases. In the present study, we first investigated the ability and mechanism of PM to reduce the degree of AngII‐induced cardiomyocyte hypertrophy in vitro and TAC‐induced cardiac hypertrophy in vivo. PM treatment of H9c2 cells suppressed the AngII‐induced expressions of hypertrophic proteins and cell size increase, and reduced the STAT3 and JAK2 phosphorylation. Furthermore, the STAT3 specific inhibitor S3I‐201 or the depletion of STAT3 expression by siRNA could alleviate AngII‐induced cardiac hypertrophy in H9c2 cells following PM treatment, while PM failed to reduce the expressions of hypertrophy‐related proteins (Collagen I, α‐SMA, TGF‐β1 and ANP) and phosphorylated STAT3 in STAT3‐overexpressing cells, suggesting that PM‐mediated protection against AngII‐induced cardiac hypertrophy depended on modulation of the STAT3 signalling axis. In addition, PM successfully prevented TAC‐induced functional and structural cardiac deficits in mice. These results clearly indicated that PM protects against pathological cardiac hypertrophy by reducing the nuclear translocation of *p*‐STAT3 and inhibiting the JAK2/STAT3 signalling pathway.

Pathological cardiac hypertrophy can gradually damage the systolic or diastolic function of the heart, which increases mortality associated with cardiovascular diseases.^2^ Therefore, identifying the novel agents for treating cardiac hypertrophy is an important goal for cardiovascular disease therapy. Traditional Chinese medicine is a collected summary of experience in long‐term clinical applications, with known advantages for treating chronic diseases. It is regarded as an important resource for discovering new active compounds.^29^ Increasing evidence has confirmed that traditional Chinese medicine is beneficial for alleviating and reversing myocardial hypertrophy, such as tanshinone IIA from Salvia miltiorrhizae Radix *et* Rhizoma, ginsenoside Rg1 from Ginseng Radix *et* Rhizoma, and berberine from Coptidis Rhizoma.^30^, ^31^, ^32^ Accordingly, natural products may provide a new direction for developing anti‐hypertrophic drugs. Our previous research has shown that PM from Periplocae cortex could be absorbed into the blood of rats and reduced ISO‐induced myocardial hypertrophy.^33^, ^34^ Therefore, PM is considered a potential therapeutic compound to alleviate cardiac hypertrophy. In order to develop this active compound, the efficacy and mechanism of PM in improving cardiac hypertrophy was investigated. In this experiment, AngII induced an enlargement of cardiomyocyte surface area accompanied with increased protein levels of Collagen I, α‐SMA, TGF‐β1 and ANP, and TAC caused interstitial fibrosis and hypertrophy in mice, which was consistent with other reports.^35^, ^36^ PM effectively reduced myocardial hypertrophy caused by AngII and TAC, manifested by decreased cell surface area and reduced expressions of hypertrophy‐related markers (Collagen I, α‐SMA, TGF‐β1 and ANP), implying that PM is a bioactive compound that improves myocardial hypertrophy and is worth further exploration.

It is well‐established that the JAK/STAT signalling pathway is widely involved in important signal transduction during pathological processes such as cardiac hypertrophy and fibrosis.^17^ JAK is a series of tyrosine kinases comprising JAK1, JAK2, JAK3 and JAK4.^37^ As the direct substrate of JAK, STAT sends signals to the nucleus and regulates the expression of specific genes.^17^, ^38^ The STAT family comprises seven known STAT types, including STAT1, STAT2, STAT3, STAT4, STAT5A, STAT5B and STAT6.^17^, ^39^ Among them, STAT3 is considered a key regulator in the process of hypertrophic growth.^14^, ^21^ In the present study, AngII obviously up‐regulated the protein expressions of *p*‐STAT3 and *p*‐JAK2 and increased the level of *p*‐STAT3 nuclear translocation, while PM down‐regulated all these changes, suggesting that PM afforded protection against AngII‐induced myocardial hypertrophy by regulating the JAK2/STAT3 signalling pathway. More importantly, STAT3 signalling was significantly inhibited in AngII‐induced hypertrophic conditions following PM, S3I‐201 and specific STAT3 siRNA treatment, whereas the specific overexpression of RNA could weaken the therapeutic effect of PM. These data revealed that PM exerted the anti‐hypertrophic effect by inhibiting the STAT3 signalling pathway. Based on the results of molecular virtual docking of PM and STAT3 (Figure S3), along with the luciferase activity (Figure S2) and the effect of PM on STAT3 expression in vitro, we postulated that PM exhibited potential as a partial STAT3 inhibitor, warranting further verification. In addition, although the selective STAT3 inhibitor, S3I‐201, was effective in reducing STAT3 phosphorylation and anti‐myocardial hypertrophy at the cellular level, it displayed a weak ability to inhibit myocardial hypertrophy in animal experiments. This finding was inconsistent with a previous report.^14^ The discrepancy may be attributed to the different methods to induce cardiac hypertrophy in vivo. We used TAC to induce cardiac hypertrophy in mice, while AngII was used to induce cardiac hypertrophy in the previous report.^14^ Different models may induce different mechanisms and drug effects, which need to be confirmed in future investigations.

## 5 CONCLUSION

**FIGURE 6:**
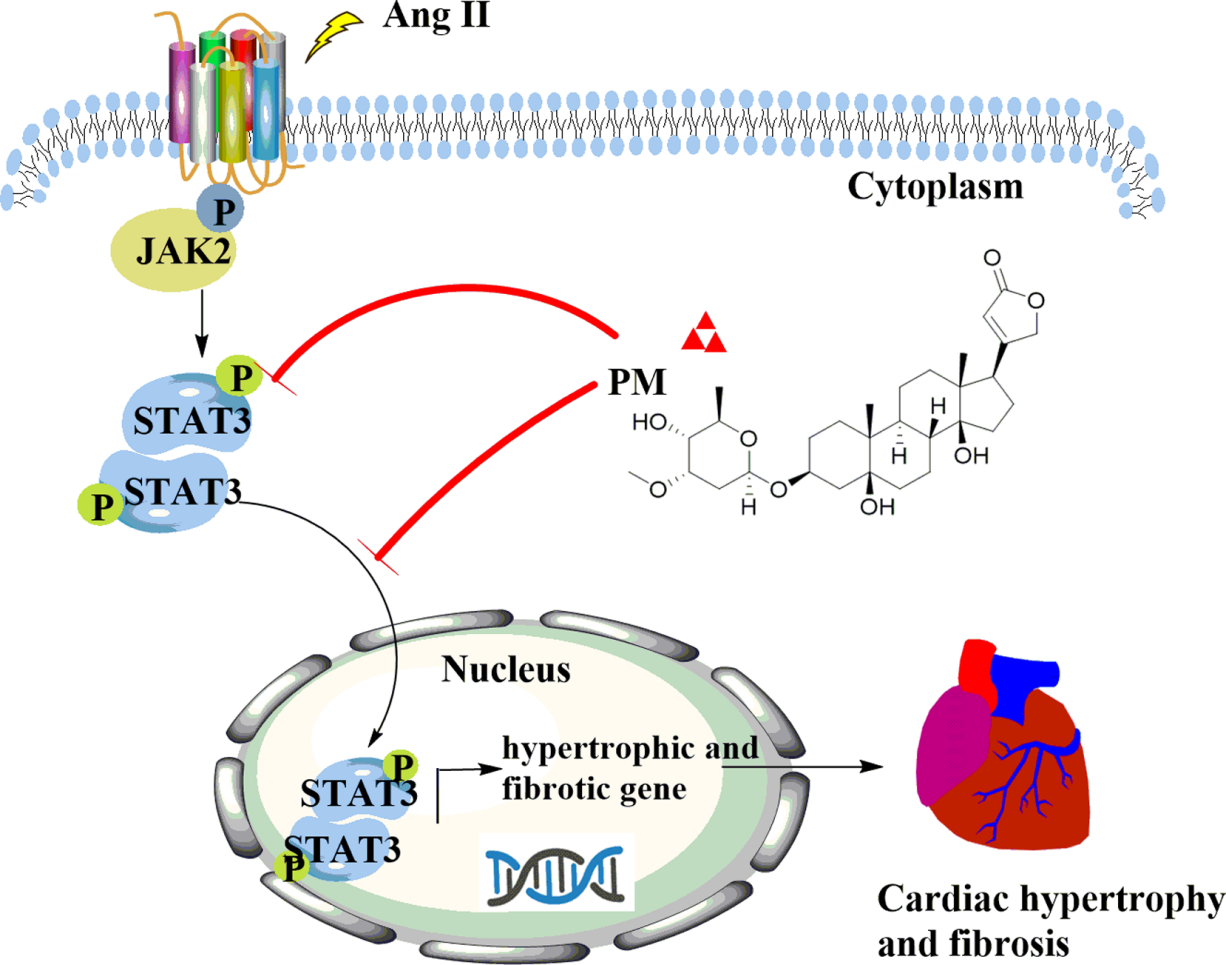
Schematic mechanism of periplocymarin (PM) in the treatment of pathological cardiac hypertrophy

In summary, our study provides evidence that PM protects against pathological cardiac hypertrophy by inhibiting levels of the STAT3 phosphorylation and nuclear translocation to suppress the activation of JAK2/STAT3 signalling pathway (Figure 6). However, the properties and clinical application of PM still require further research for the development as a potential new lead compound to treat pathological cardiac hypertrophy.

## CONFLICT OF INTEREST

All authors clearly state that they have no conflict of interest.

## AUTHOR CONTRIBUTIONS

**Cailian Fan:** Data curation (equal); Investigation (equal); Project administration (equal); Resources (equal); Validation (equal); Visualization (equal); Writing – original draft (equal). **Sui Liang:** Investigation (equal); Validation (equal); Writing – original draft (equal). **Mengnan Ye:** Data curation (equal); Investigation (equal); Methodology (equal); Validation (equal). **Wanjun Cai:** Data curation (equal); Methodology (equal); Validation (equal); Visualization (equal). **Miao Chen:** Data curation (equal); Investigation (equal); Methodology (equal); Validation (equal). **Yunlong Hou:** Data curation (equal); Methodology (equal); Project administration (equal); Writing – review & editing (equal). **Jun Guo:** Investigation (equal); Project administration (equal); Supervision (equal); Writing – review & editing (equal). **Yi Dai:** Investigation (equal); Project administration (equal); Writing – original draft (equal); Writing – review & editing (equal).

## Supporting information

Figure S1‐S5

Supplementary Material

Supplementary Material

## References

[1] Marian AJ . Molecular genetic basis of hypertrophic cardiomyopathy. Circ Res. 2021;128:1533‐1553. 33983830 10.1161/CIRCRESAHA.121.318346 PMC8127615

[2] Lerchenmüller C , Rabolli CP , Yeri A , et al. CITED4 protects against adverse remodeling in response to physiological and pathological stress. Circ Res. 2020;127:631‐646. 32418505 10.1161/CIRCRESAHA.119.315881 PMC7725361

[3] Gibb AA , Hill BG . Metabolic coordination of physiological and pathological cardiac remodeling. Circ Res. 2018;123:107‐128. 29929976 10.1161/CIRCRESAHA.118.312017 PMC6023588

[4] Vanhoutte D , Schips TG , Vo A , et al. Thbs1 induces lethal cardiac atrophy through PERK‐ATF4 regulated autophagy. Nat Commun. 2021;12:3928. 34168130 10.1038/s41467-021-24215-4 PMC8225674

[5] Bacmeister L , Schwarzl M , Warnke S , et al. Inflammation and fibrosis in murine models of heart failure. Basic Res Cardiol. 2019;114:19. 30887214 10.1007/s00395-019-0722-5

[6] Liu X , Shi GP , Guo JL . Innate immune cells in pressure overload‐induced cardiac hypertrophy and remodeling. Front Cell Dev Biol. 2021;9:659666. 34368120 10.3389/fcell.2021.659666 PMC8343105

[7] Yang L , Deng JX , Ma WX , et al. Ablation of lncRNA Miat attenuates pathological hypertrophy and heart failure. Theranostics. 2021;11:7995‐8007. 34335976 10.7150/thno.50990 PMC8315059

[8] Curtiss C , Cohn JN , Vrobel T , Franciosa IA . Role of the renin‐angiotensin system in the systemic vasoconstriction of chronic congestive heart failure. Circulation. 1978;58:763‐770. 699245 10.1161/01.cir.58.5.763

[9] Shen YJ , Wang X , Yuan RS , et al. Prostaglandin E1 attenuates Ang II‐induced cardiac hypertrophy *via* EP3 receptor activation and Netrin‐1 upregulation. J Mol Cell Cardiol. 2021;159:91‐104. 34147480 10.1016/j.yjmcc.2021.06.009

[10] García‐Martín A , Navarrete C , Garrido‐Rodríguez M , et al. EHP‐101 alleviates angiotensin II‐induced fibrosis and inflammation in mice. Biomed Pharmacother. 2021;142:112007. 34385107 10.1016/j.biopha.2021.112007

[11] Sarkar S , Vellaichamy E , Young D , Sen S . Influence of cytokines and growth factors in ANG II‐mediated collagen upregulation by fibroblasts in rats: role of myocytes. Am J Physiol Heart Circ Physiol. 2001;287:H107‐H117. 10.1152/ajpheart.00763.2003 15059775

[12] An DQ , Zeng QC , Zhang PJ , et al. Alpha‐ketoglutarate ameliorates pressure overload‐induced chronic cardiac dysfunction in mice. Redox Biol. 2020;46:102088. 10.1016/j.redox.2021.102088 PMC8353361 34364218

[13] Ji YY , Wang ZD , Li ZF , Li K , Le XF , Zhang T . Angiotensin II induces angiogenic factors production partly *via* AT1/JAK2/STAT3/SOCS3 signaling pathway in MHCC97H cells. Cell Physiol Biochem. 2021;29:863‐874. 10.1159/000171034 22613986

[14] Ye SJ , Luo W , Khan ZA , et al. Celastrol attenuates angiotensin II‐induced cardiac remodeling by targeting STAT3. Circ Res. 2020;126:1007‐1023. 32098592 10.1161/CIRCRESAHA.119.315861

[15] Fasouli ES , Katsantoni E . JAK‐STAT in early hematopoiesis and leukemia. Front Cell Dev Biol. 2021;9:669363. 34055801 10.3389/fcell.2021.669363 PMC8160090

[16] Ou A , Ott M , Fang DX , Heimberger AB . The role and therapeutic targeting of JAK/STAT signaling in glioblastoma. Cancers. 2021;13:437. 33498872 10.3390/cancers13030437 PMC7865703

[17] Chiara B , Romana MF , Sara G , Peter L , Raffaele DC . The JAK‐STAT pathway: an emerging target for cardiovascular disease in rheumatoid arthritis and myeloproliferative neoplasms. Eur Heart J. 2021;42(42):4389‐4400. 34343257 10.1093/eurheartj/ehab447 PMC8572559

[18] Yerabolu D , Weiss A , Kojonazarov B , et al. Targeting JAK‐STAT signaling in experimental pulmonary hypertension. Am J Respir Cell Mol. 2020;64(64):100‐114. 10.1165/rcmb.2019-0431OC 33052714

[19] Shi JJ , Wei L . Regulation of JAK/STAT signalling by SOCS in the myocardium. Cardiovasc Res. 2012;96:345‐347. 23090607 10.1093/cvr/cvs321

[20] Wu CH , Ge J , Yang M , et al. Resveratrol protects human nucleus pulposus cells from degeneration by blocking IL‐6/JAK/STAT3 pathway. Euro J Med Res. 2021;26(1):81. 10.1186/s40001-021-00555-1 PMC8320225 34321087

[21] Yang YZ , Cheng X , Tang SQ , Xia ZY . Interleukin‐9 aggravates isoproterenol‐induced heart failure by activating signal transducer and activator of transcription 3 signalling. Can J Cardiol. 2020;36:1770‐1781. 32621886 10.1016/j.cjca.2020.01.011

[22] Kunisada K , Negoro S , Tone E , et al. Signal transducer and activator of transcription 3 in the heart transduces not only a hypertrophic signal but a protective signal against doxorubicin‐induced cardiomyopathy. Proc Natl Acad Sci USA. 2000;97:315‐319. 10618415 10.1073/pnas.97.1.315 PMC26660

[23] Cazzaniga G , Mori M , Chiarelli LR , Gelain A , Meneghetti F , Villa S . Natural products against key mycobacterium tuberculosis enzymatic targets: emerging opportunities for drug discovery. Eur J Med Chem. 2021;224:113732. 34399099 10.1016/j.ejmech.2021.113732

[24] Lohberger B , Wagner S , Wohlmuther J , et al. Periplocin, the most anti‐proliferative constituent of Periploca sepium, specifically kills liposarcoma cells by death receptor mediated apoptosis. Phytomedicine. 2018;51:162‐170. 30466613 10.1016/j.phymed.2018.10.008

[25] Bloise E , Braca A , Tommasi ND , Belisario MA . Pro‐apoptotic and cytostatic activity of naturally occurring cardenolides. Cancer Chemother Pharmacol. 2009;64:793‐802. 19184018 10.1007/s00280-009-0929-5

[26] Yun WJ , Qian L , Yuan RQ , Xu H . Periplocymarin protects against myocardial fibrosis induced by β‐adrenergic activation in mice. Biomed Pharmacother. 2021;139:111562. 33839492 10.1016/j.biopha.2021.111562

[27] Li S , Zhu ZX , Xue M , et al. Fibroblast growth factor 21 protects the heart from angiotensin II‐induced cardiac hypertrophy and dysfunction *via* SIRT1. Biochim Biophys Acta Mol Basis Dis. 2019;1865:1241‐1252. 30677512 10.1016/j.bbadis.2019.01.019

[28] Gao S , Park BM , Cha SA , Park WH , Park BH , Kim SH . Angiotensin AT2 receptor agonist stimulates high stretch induced‐ ANP secretion *via* PI3K/NO/sGC/PKG/pathway. Peptides. 2013;47:36‐44. 23791669 10.1016/j.peptides.2013.06.008

[29] Liu W , Wang HL , Zhu B , et al. An activity‐integrated strategy of the identification, screening and determination of potential neuraminidase inhibitors from Radix Scutellariae. PLoS One. 2017;12:e0175751. 28486473 10.1371/journal.pone.0175751 PMC5423611

[30] Gao S , Li LY , Li L , et al. Effects of the combination of tanshinone IIA and puerarin on cardiac function and inflammatory response in myocardial ischemia mice. J Mol Cell Cardiol. 2019;137:59‐70. 31629735 10.1016/j.yjmcc.2019.09.012

[31] Zhang YJ , Zhang XL , Li HM , et al. The Ginsenoside Rg1 pevents transverse aortic constriction‐induced left ventricular hypertrophy and cardiac dysfunction by inhibiting fibrosis and enhancing angiogenesis. J Cardiovasc Pharmacol. 2013;62:50‐57. 23846802 10.1097/FJC.0b013e31828f8d45

[32] Chen X , Jiang XZ , Cheng CF , et al. Berberine attenuates cardiac hypertrophy through inhibition of mTOR signaling pathway. Cardiovasc Drugs Ther. 2020;34:463‐473. 32394178 10.1007/s10557-020-06977-z

[33] Li ZT , Zhang FX , Fan CL , et al. Discovery of potential Q‐marker of traditional Chinese medicine based on plant metabolomics and network pharmacology: Periplocae cortex as an example. Phytomedicine. 2021;85:153535. 33819766 10.1016/j.phymed.2021.153535

[34] He LL , Liu YH , Yang KF , et al. The discovery of Q‐markers of Qiliqiangxin Capsule, a traditional Chinese medicine prescription in the treatment of chronic heart failure, based on a novel strategy of multi‐dimensional "radar chart" mode evaluation. Phytomedicine. 2020;82:153443. 33429210 10.1016/j.phymed.2020.153443

[35] Kerp H , Hönes GS , Tolstik E , et al. Protective effects of thyroid hormone deprivation on progression of maladaptive cardiac hypertrophy and heart failure. Front Cardiovasc Med. 2021;8:683522. 34395557 10.3389/fcvm.2021.683522 PMC8363198

[36] Ye SJ , Lin K , Wu GJ , et al. Toll‐like receptor 2 signaling deficiency in cardiac cells ameliorates Ang II‐induced cardiac inflammation and remodeling. Transl Res. 2021;233:62‐76. 33652137 10.1016/j.trsl.2021.02.011

[37] Zhao LL , Wu DW , Sang MR , Xu YM , Liu ZG , Wu QN . Stachydrine ameliorates isoproterenol‐induced cardiac hypertrophy and fibrosis by suppressing inflammation and oxidative stress through inhibiting NF‐κB and JAK/STAT signaling pathways in rats. Int Immunopharmacol. 2017;48:102‐109. 28499193 10.1016/j.intimp.2017.05.002

[38] Xue X , Jungles K , Onder G , Samhoun J , Hardiman KM . HIF‐3α1 promotes colorectal tumor cell growth by activation of JAK‐STAT3 signaling. Oncotarget. 2016;7:11567‐11576. 26871465 10.18632/oncotarget.7272 PMC4905494

[39] Zhong Z , Wen Z , Darnell JE . Stat3: a STAT family member activated by tyrosine phosphorylation in response to epidermal growth factor and interleukin‐6. Science. 1994;264:95‐98. 8140422 10.1126/science.8140422

