# Supplementary material for: Periplocymarin alleviates pathological cardiac hypertrophy via inhibiting the JAK2/STAT3 signalling pathway
Source: J Cell Mol Med. 2022 Apr 1;26(9):2607–19. doi: 10.1111/jcmm.17267 (PMC9077305; doi:10.1111/jcmm.17267)
Supplement: Supplementary file 1 — Figure S1‐S5 [file JCMM-26-2607-s001.docx]

***Supplementary materials***

**Periplocymarin alleviates pathological cardiac hypertrophy *via* inhibiting the JAK2/STAT3 signaling pathway**

**1. The plasmid banding pattern of Stat3(NM_012747.2)-pcDNA3.1-3xFlag-CV1.**


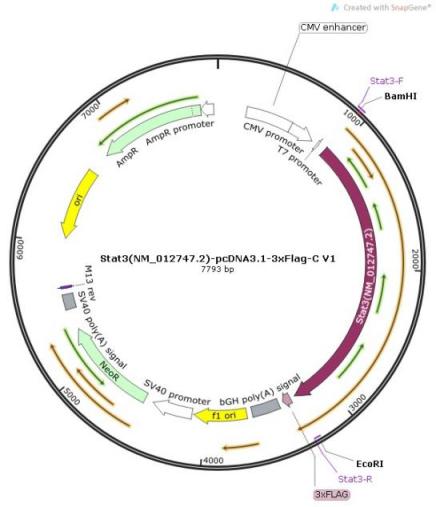


**Fig. S1** The plasmid banding patterns of Stat3(NM_012747.2)-pcDNA3.1-3xFlag-CV1

1. **Overexpression plasmid sequence of Stat3(NM_012747.2)-pcDNA3.1-3xFlag-CV**

GACGGATCGGGAGATCTCCCGATCCCCTATGGTGCACTCTCAGTACAATCTGCTCTGATGCCGCATAGTTAAGCCAGTATCTGCTCCCTGCTTGTGTGTTGGAGGTCGCTGAGTAGTGCGCGAGCAAAATTTAAGCTACAACAAGGCAAGGCTTGACCGACAATTGCATGAAGAATCTGCTTAGGGTTAGGCGTTTTGCGCTGCTTCGCGATGTACGGGCCAGATATACGCGTTGACATTGATTATTGACTAGTTATTAATAGTAATCAATTACGGGGTCATTAGTTCATAGCCCATATATGGAGTTCCGCGTTACATAACTTACGGTAAATGGCCCGCCTGGCTGACCGCCCAACGACCCCCGCCCATTGACGTCAATAATGACGTATGTTCCCATAGTAACGCCAATAGGGACTTTCCATTGACGTCAATGGGTGGAGTATTTACGGTAAACTGCCCACTTGGCAGTACATCAAGTGTATCATATGCCAAGTACGCCCCCTATTGACGTCAATGACGGTAAATGGCCCGCCTGGCATTATGCCCAGTACATGACCTTATGGGACTTTCCTACTTGGCAGTACATCTACGTATTAGTCATCGCTATTACCATGGTGATGCGGTTTTGGCAGTACATCAATGGGCGTGGATAGCGGTTTGACTCACGGGGATTTCCAAGTCTCCACCCCATTGACGTCAATGGGAGTTTGTTTTGGCACCAAAATCAACGGGACTTTCCAAAATGTCGTAACAACTCCGCCCCATTGACGCAAATGGGCGGTAGGCGTGTACGGTGGGAGGTCTATATAAGCAGAGCTCTCTGGCTAACTAGAGAACCCACTGCTTACTGGCTTATCGAAATTAATACGACTCACTATAGGGAGACCCAAGCTGGCTAGCGTTTAAACTTAAGCTTGGTACCGAGCTCGGATCCGCCACCatggctcagtggaaccagctccagcagctggacacgcgctacctggagcagcttcatcagctgtacagcgatagcttccccatggagctgcggcagttcctggcgccttggattgagagccaagattgggcatatgcagccagcaaagagtcacacgccactctggtgtttcataacctcttgggcgagatcgaccagcagtatagccgattcctgcaggagtccaatgtcctctatcagcacaacctgcgaagaatcaagcagttcctgcagagcaggtatcttgagaagccaatggaaattgcccggattgtggcccgatgcctgtgggaagagtctcgcctcctccagacggcagccacggcagcccagcaagggggccaggccaaccaccccacagctgccgtagtgacggagaagcagcagatgctggaacagcatcttcaggatgtccggaagcgtgtgcaggatctagaacagaaaatgaaagtggtggagaatctccaggatgactttgatttcaactataaaaccctcaagagtcaaggagacatgcaggatctgaatggaaacaaccagtctgtgaccagacagaagatgcagcagctggagcagatgctcacggccctggaccagatgcggaggagcatcgtgagcgagctggcagggctcttgtcagcaatggagtacgtgcagaagacactgaccgatgaagagctggctgactggaagaggcggcagcagatagcgtgcatcggaggccctcccaacatctgcctggaccgtctggaaaactggataacttcattagcagaatctcaacttcagacccgccaacaaattaagaaactggaggagctgcagcagaaagtgtcctacaagggggaccctattgtgcagcaccggccaatgctggaggagaggatcgtggatctgttcagaaacttaatgaagagtgccttcgtggtggagcggcagccctgtatgcccatgcacccggaccggcccttagtcatcaagactggtgtccagtttaccacaaaagtcaggttgctggtcaaatttcctgagttgaattatcagcttaaaattaaagtgtgcattgataaggactctggggatgttgctgccctcagagggtctcggaaatttaacattctgggcacgaacacaaaggtgatgaacatggaggagtccaacaacggcagcctgtctgcagagttcaagcacctgaccctgagggagcagagatgtgggaatgggggccgtgccaattgtgatgcctccttgattgtcactgaggagctgcacctgatcacctttgagacagaggtgtaccaccaaggtctcaagatcgacctagagacccactccttgccagtcgtggtgatctccaacatctgtcagatgcctaatgcttgggcatcaatcctgtggtataacatgctgaccaataaccccaagaacgtgaacttcttcactaagcctccgattggaacctgggaccaagtggccgaggtgctgagctggcagttctcgtccaccaccaagcgagggctgagcatcgagcagctgaccacgctggccgagaagctcttagggcctggtgtgaactactcagggtgtcagatcacatgggctaagttttgcaaagaaaacatggccggcaagggcttctcgttctgggtctggctagacaatatcatcgaccttgtgaaaaagtatatcttggccctttggaatgaagggtacatcatgggtttcatcagcaaggagcgggagagggccatcctaagcacaaagcccccgggcaccttcctgctgcggttcagtgagagcagcaaggaaggaggggtcactttcacttgggtggaaaaggacatcagtggcaagacccagatccagtctgtagaaccatataccaagcagcagctgaacaacatgtcatttgctgaaatcatcatgggctataagatcatggacgctaccaacatcctggtatccccactggtctacctctaccctgacattcccaaggaggaggcattcggaaagtattgtcgccccgagagccaggagcaccctgaagctgacccaggtagtgctgccccttacctgaagaccaagttcatctgtgtgacaccaacgacctgcagcaataccattgacctgccgatgtccccccgcactttagattcattgatgcagtttggaaataacggggaaggcgctgagccctcagcaggagggcagtttgagtcgctcacgtttgacatggatctgacctcggagtgtgctacctccccgatgGAATTCTGCAGATATCCAGCACAGTGGCGGCCGCTCGAGGACTACAAAGACCATGACGGTGATTATAAAGATCATGACATCGACTACAAGGATGACGATGACAAGTAGTGAGGGCCCGTTTAAACCCGCTGATCAGCCTCGACTGTGCCTTCTAGTTGCCAGCCATCTGTTGTTTGCCCCTCCCCCGTGCCTTCCTTGACCCTGGAAGGTGCCACTCCCACTGTCCTTTCCTAATAAAATGAGGAAATTGCATCGCATTGTCTGAGTAGGTGTCATTCTATTCTGGGGGGTGGGGTGGGGCAGGACAGCAAGGGGGAGGATTGGGAAGACAATAGCAGGCATGCTGGGGATGCGGTGGGCTCTATGGCTTCTGAGGCGGAAAGAACCAGCTGGGGCTCTAGGGGGTATCCCCACGCGCCCTGTAGCGGCGCATTAAGCGCGGCGGGTGTGGTGGTTACGCGCAGCGTGACCGCTACACTTGCCAGCGCCCTAGCGCCCGCTCCTTTCGCTTTCTTCCCTTCCTTTCTCGCCACGTTCGCCGGCTTTCCCCGTCAAGCTCTAAATCGGGGGCTCCCTTTAGGGTTCCGATTTAGTGCTTTACGGCACCTCGACCCCAAAAAACTTGATTAGGGTGATGGTTCACGTAGTGGGCCATCGCCCTGATAGACGGTTTTTCGCCCTTTGACGTTGGAGTCCACGTTCTTTAATAGTGGACTCTTGTTCCAAACTGGAACAACACTCAACCCTATCTCGGTCTATTCTTTTGATTTATAAGGGATTTTGCCGATTTCGGCCTATTGGTTAAAAAATGAGCTGATTTAACAAAAATTTAACGCGAATTAATTCTGTGGAATGTGTGTCAGTTAGGGTGTGGAAAGTCCCCAGGCTCCCCAGCAGGCAGAAGTATGCAAAGCATGCATCTCAATTAGTCAGCAACCAGGTGTGGAAAGTCCCCAGGCTCCCCAGCAGGCAGAAGTATGCAAAGCATGCATCTCAATTAGTCAGCAACCATAGTCCCGCCCCTAACTCCGCCCATCCCGCCCCTAACTCCGCCCAGTTCCGCCCATTCTCCGCCCCATGGCTGACTAATTTTTTTTATTTATGCAGAGGCCGAGGCCGCCTCTGCCTCTGAGCTATTCCAGAAGTAGTGAGGAGGCTTTTTTGGAGGCCTAGGCTTTTGCAAAAAGCTCCCGGGAGCTTGTATATCCATTTTCGGATCTGATCAAGAGACAGGATGAGGATCGTTTCGCATGATTGAACAAGATGGATTGCACGCAGGTTCTCCGGCCGCTTGGGTGGAGAGGCTATTCGGCTATGACTGGGCACAACAGACAATCGGCTGCTCTGATGCCGCCGTGTTCCGGCTGTCAGCGCAGGGGCGCCCGGTTCTTTTTGTCAAGACCGACCTGTCCGGTGCCCTGAATGAACTGCAGGACGAGGCAGCGCGGCTATCGTGGCTGGCCACGACGGGCGTTCCTTGCGCAGCTGTGCTCGACGTTGTCACTGAAGCGGGAAGGGACTGGCTGCTATTGGGCGAAGTGCCGGGGCAGGATCTCCTGTCATCTCACCTTGCTCCTGCCGAGAAAGTATCCATCATGGCTGATGCAATGCGGCGGCTGCATACGCTTGATCCGGCTACCTGCCCATTCGACCACCAAGCGAAACATCGCATCGAGCGAGCACGTACTCGGATGGAAGCCGGTCTTGTCGATCAGGATGATCTGGACGAAGAGCATCAGGGGCTCGCGCCAGCCGAACTGTTCGCCAGGCTCAAGGCGCGCATGCCCGACGGCGAGGATCTCGTCGTGACCCATGGCGATGCCTGCTTGCCGAATATCATGGTGGAAAATGGCCGCTTTTCTGGATTCATCGACTGTGGCCGGCTGGGTGTGGCGGACCGCTATCAGGACATAGCGTTGGCTACCCGTGATATTGCTGAAGAGCTTGGCGGCGAATGGGCTGACCGCTTCCTCGTGCTTTACGGTATCGCCGCTCCCGATTCGCAGCGCATCGCCTTCTATCGCCTTCTTGACGAGTTCTTCTGAGCGGGACTCTGGGGTTCGAAATGACCGACCAAGCGACGCCCAACCTGCCATCACGAGATTTCGATTCCACCGCCGCCTTCTATGAAAGGTTGGGCTTCGGAATCGTTTTCCGGGACGCCGGCTGGATGATCCTCCAGCGCGGGGATCTCATGCTGGAGTTCTTCGCCCACCCCAACTTGTTTATTGCAGCTTATAATGGTTACAAATAAAGCAATAGCATCACAAATTTCACAAATAAAGCATTTTTTTCACTGCATTCTAGTTGTGGTTTGTCCAAACTCATCAATGTATCTTATCATGTCTGTATACCGTCGACCTCTAGCTAGAGCTTGGCGTAATCATGGTCATAGCTGTTTCCTGTGTGAAATTGTTATCCGCTCACAATTCCACACAACATACGAGCCGGAAGCATAAAGTGTAAAGCCTGGGGTGCCTAATGAGTGAGCTAACTCACATTAATTGCGTTGCGCTCACTGCCCGCTTTCCAGTCGGGAAACCTGTCGTGCCAGCTGCATTAATGAATCGGCCAACGCGCGGGGAGAGGCGGTTTGCGTATTGGGCGCTCTTCCGCTTCCTCGCTCACTGACTCGCTGCGCTCGGTCGTTCGGCTGCGGCGAGCGGTATCAGCTCACTCAAAGGCGGTAATACGGTTATCCACAGAATCAGGGGATAACGCAGGAAAGAACATGTGAGCAAAAGGCCAGCAAAAGGCCAGGAACCGTAAAAAGGCCGCGTTGCTGGCGTTTTTCCATAGGCTCCGCCCCCCTGACGAGCATCACAAAAATCGACGCTCAAGTCAGAGGTGGCGAAACCCGACAGGACTATAAAGATACCAGGCGTTTCCCCCTGGAAGCTCCCTCGTGCGCTCTCCTGTTCCGACCCTGCCGCTTACCGGATACCTGTCCGCCTTTCTCCCTTCGGGAAGCGTGGCGCTTTCTCATAGCTCACGCTGTAGGTATCTCAGTTCGGTGTAGGTCGTTCGCTCCAAGCTGGGCTGTGTGCACGAACCCCCCGTTCAGCCCGACCGCTGCGCCTTATCCGGTAACTATCGTCTTGAGTCCAACCCGGTAAGACACGACTTATCGCCACTGGCAGCAGCCACTGGTAACAGGATTAGCAGAGCGAGGTATGTAGGCGGTGCTACAGAGTTCTTGAAGTGGTGGCCTAACTACGGCTACACTAGAAGAACAGTATTTGGTATCTGCGCTCTGCTGAAGCCAGTTACCTTCGGAAAAAGAGTTGGTAGCTCTTGATCCGGCAAACAAACCACCGCTGGTAGCGGTTTTTTTGTTTGCAAGCAGCAGATTACGCGCAGAAAAAAAGGATCTCAAGAAGATCCTTTGATCTTTTCTACGGGGTCTGACGCTCAGTGGAACGAAAACTCACGTTAAGGGATTTTGGTCATGAGATTATCAAAAAGGATCTTCACCTAGATCCTTTTAAATTAAAAATGAAGTTTTAAATCAATCTAAAGTATATATGAGTAAACTTGGTCTGACAGTTACCAATGCTTAATCAGTGAGGCACCTATCTCAGCGATCTGTCTATTTCGTTCATCCATAGTTGCCTGACTCCCCGTCGTGTAGATAACTACGATACGGGAGGGCTTACCATCTGGCCCCAGTGCTGCAATGATACCGCGAGACCCACGCTCACCGGCTCCAGATTTATCAGCAATAAACCAGCCAGCCGGAAGGGCCGAGCGCAGAAGTGGTCCTGCAACTTTATCCGCCTCCATCCAGTCTATTAATTGTTGCCGGGAAGCTAGAGTAAGTAGTTCGCCAGTTAATAGTTTGCGCAACGTTGTTGCCATTGCTACAGGCATCGTGGTGTCACGCTCGTCGTTTGGTATGGCTTCATTCAGCTCCGGTTCCCAACGATCAAGGCGAGTTACATGATCCCCCATGTTGTGCAAAAAAGCGGTTAGCTCCTTCGGTCCTCCGATCGTTGTCAGAAGTAAGTTGGCCGCAGTGTTATCACTCATGGTTATGGCAGCACTGCATAATTCTCTTACTGTCATGCCATCCGTAAGATGCTTTTCTGTGACTGGTGAGTACTCAACCAAGTCATTCTGAGAATAGTGTATGCGGCGACCGAGTTGCTCTTGCCCGGCGTCAATACGGGATAATACCGCGCCACATAGCAGAACTTTAAAAGTGCTCATCATTGGAAAACGTTCTTCGGGGCGAAAACTCTCAAGGATCTTACCGCTGTTGAGATCCAGTTCGATGTAACCCACTCGTGCACCCAACTGATCTTCAGCATCTTTTACTTTCACCAGCGTTTCTGGGTGAGCAAAAACAGGAAGGCAAAATGCCGCAAAAAAGGGAATAAGGGCGACACGGAAATGTTGAATACTCATACTCTTCCTTTTTCAATATTATTGAAGCATTTATCAGGGTTATTGTCTCATGAGCGGATACATATTTGAATGTATTTAGAAAAATAAACAAATAGGGGTTCCGCGCACATTTCCCCGAAAAGTGCCACCTGACGTC

1. **Oligos used for knockdown of genes**

SH1: GCCTTGGATTGAGAGCCAAGA

| S | CACCGGCCTTGGATTGAGAGCCAAGATTCAAGAGAtcttggctctcaatccaaggcTTTTTTG |
| --- | --- |
| A | GATCCAAAAAAGCCTTGGATTGAGAGCCAAGATCTCTTGAAtcttggctctcaatccaaggcC |

SH2：GGAACAGCATCTTCAGGATGT

| S | CACCGGGAACAGCATCTTCAGGATGTTTCAAGAGAacatcctgaagatgctgttccTTTTTTG |
| --- | --- |
| A | GATCCAAAAAAGGAACAGCATCTTCAGGATGTTCTCTTGAAacatcctgaagatgctgttccC |

SH3：GCATCAATCCTGTGGTATAAC

| S | CACCGGCATCAATCCTGTGGTATAACTTCAAGAGAgttataccacaggattgatgcTTTTTTG |
| --- | --- |
| A | GATCCAAAAAAGCATCAATCCTGTGGTATAACTCTCTTGAAgttataccacaggattgatgcC |

1. **PM inhibited IL-6-induced STAT3 expression using luciferase report.**


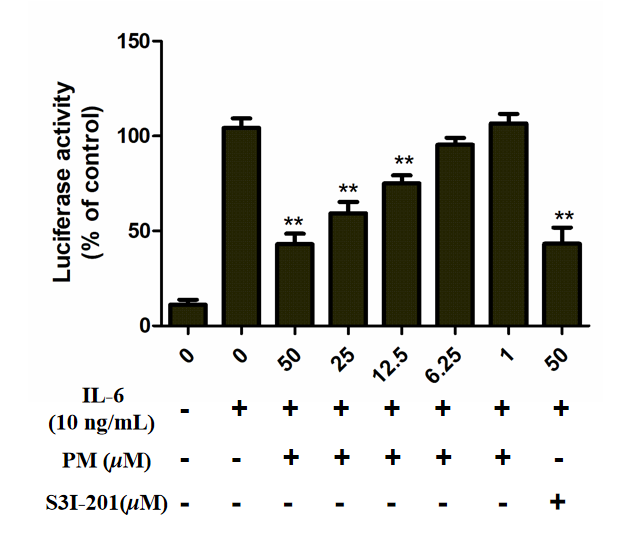


**Fig. S2** PM inhibited IL-6-induced STAT3 expression

1. **PM could bind to STAT3 by analysis of molecular docking results.**

With the help of the online tool of Swiss target prediction, the potential target STAT3 of PM was found, and then the STAT3 crystal structure 6NJS (**Fig. S3-A, B**) was obtained from the PDB database (https://www1.rcsb.org/structure/6NJS). The glide module in Schrodinger 2015 software was used for molecular docking. As shown in **Fig. S3-C** and **D**, PM could enter the active pocket of STAT3, and linked with ser 611 and ser 613 by hydrogen bond. In addition, the binding energy between PM and STAT3 was -6.311 kcal/mol, suggesting that maybe there was a good binding affinity between PM and STAT3.


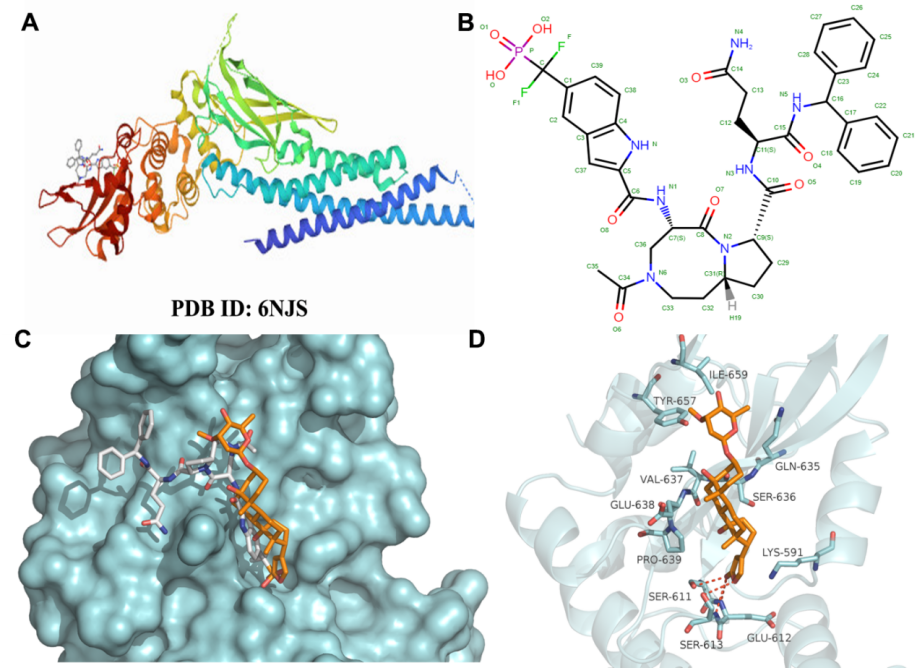


**Fig. S3** Analysis of crystal structure and binding mode of PM and STAT3

A: crystal structure of 6NJS; B: three-dimensional structure of 6NJS; C. D: docking results of PM and STAT3 crystal (6NJS).

1. **Isolation and identification of primary cardiomyocytes**

Primary cardiomyocytes from the neonatal mouse hearts were isolated. In short, 1-3-day-old new-born mice were sacrificed by decapitation. The ventricle was immediately excised and digested in ice-cold PBS (Ca^2+^ and Mg^2+^ free) with 0.125% trypsin and 0.05% type I collagenase solutions. To enrich cardiomyocytes, the cells were pre-seeded for 1.5h to remove non-cardiomyocytes. Primary cardiomyocytes were cultured on collagen-coated tissue culture dishes in cardiomyocyte culture medium containing 10% FBS. All cells were kept in a 37℃, 5% CO_2_ incubator. After primary neonatal mouse cardiomyocytes were extracted and cultured for 48h, the morphology of cells was observed and recorded by microscope (**Fig.S4A**). The primary cardiomyocytes were identified by immunofluorescence staining using α-actin antibody (**Fig.S4B**).


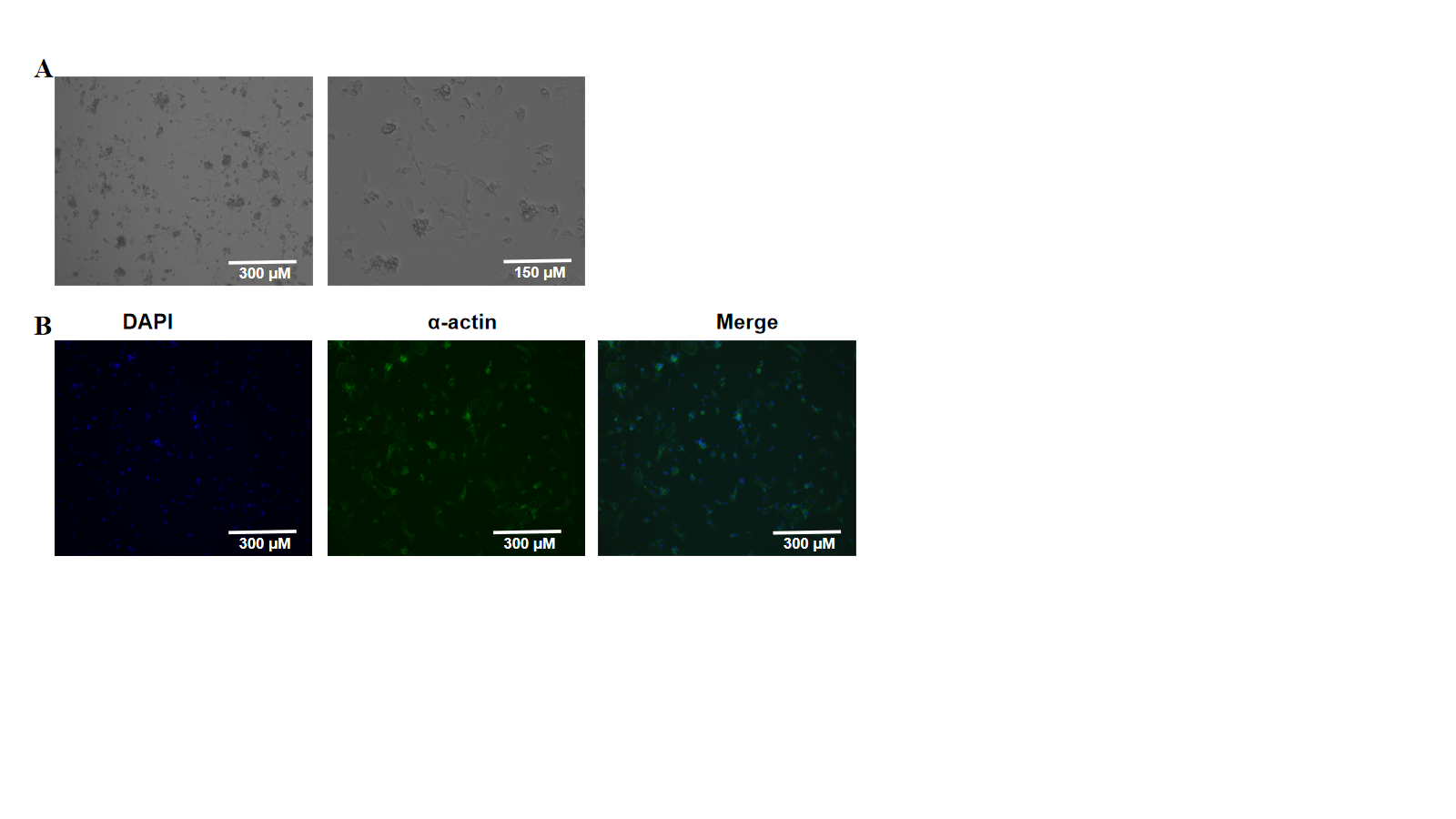


**Fig. S4** Isolation and identification of primary cardiomyocytes from the mouse neonatal heart

1. **The effect of PM on AngⅡ-induced hypertrophy of primary cardiomyocyte**

Primary cardiomyocytes were pre-treated with different concentrations of PM (12.5, 25, 50 *μ*M) 3h, and then submitted to 1 *μ*M AngⅡ for 24 h. Results from **Fig. S5A-D** displayed that 25, 50 *μ*M PM effectively down-regulated the levels of hypertrophic proteins (Collagen I, TGF-β1 and ANP, *p* < 0.05). In addition, the phosphorylation of STAT3(Tyr705) and JAK2(Tyr1007/1008) were evaluated. In primary cardiomyocytes, AngⅡ treated increased the expressions of *p*-JAK2 (Tyr1007/1008)/JAK2 and p-STAT3 (Tyr705)/STAT3, while 25, 50 *μ*M PM significantly down-regulated the ratios of them (*p* < 0.05, **Fig. S5A, E, F**). Above results from primary mouse neonatal cardiomyocytes were consistent with those obtained by H9c2 cells. All data further confirmed that PM could protect AngⅡ-induced cardiac hypertrophy.


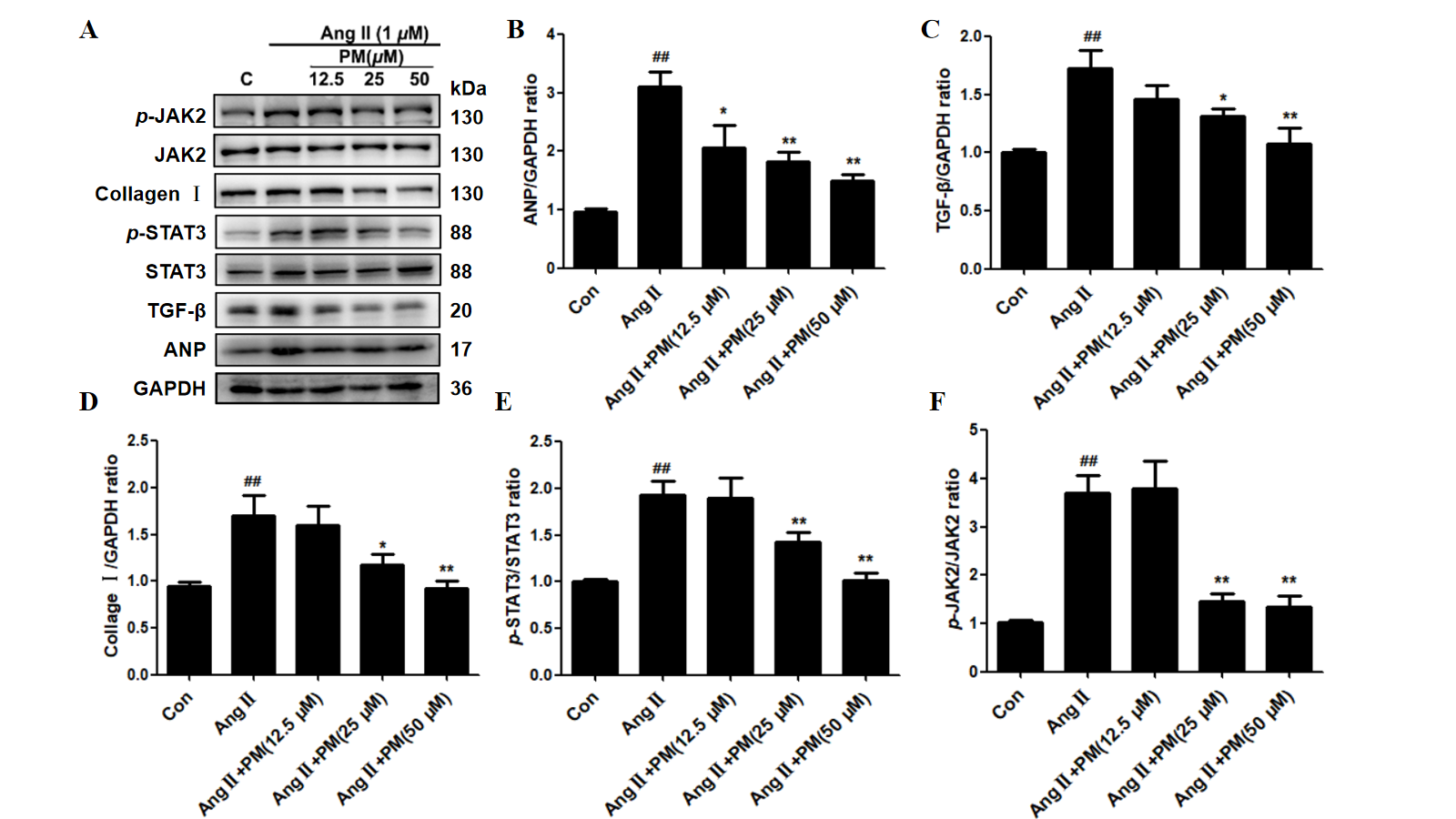


**Fig.S5** The effect of PM on AngⅡ-induced hypertrophy of primary cardiomyocyte

A: The expressions of ANP, TGF-β, Collagen I, *p*-JAK2 (Tyr1007+1008), JAK2, *p*-STAT3 (Tyr705) and STAT3 were detected by western blot. B-F: Relative protein levels of ANP/GADPH, TGF-β/GADPH, Collage Ⅰ/GAPDH, *p*-STAT3/STAT3 and p-JAK2/JAK2 in different groups were measured by Image J. ^##^*p* < 0.01 *vs*. control group. ^*^*p* < 0.05, ^**^*p* < 0.01 *vs.* AngII group. Data were presented as the mean ± SD, n=3.

1. **Abbreviations**

AngⅡ, angiotensinⅡ; BW, body weight; ANP, atrial natriuretic peptide; TL, tibia length; DAPI, 4',6-diamidino-2-phenylindole; FITC-Phalloidin, fluorescein isothiocyanate-phalloidin; GAPDH, glyceraldehyde-3-phosphate dehydrogenase; FBS, fetal bovine serum; HE staining, hematoxylin-eosin staining; HW, heart weight; JAK2, janus kinase 2; SD, Standard deviation; STAT3, signal transducer and activator of transcription 3; S3I-201, STAT3 inhibitor VI; TGF-β1, transforming growth factor-β1; Val, valsartan; α-SMA, α-smooth muscle actin.
