# Supplementary material for: Periplocymarin alleviates pathological cardiac hypertrophy via inhibiting the JAK2/STAT3 signalling pathway
Source: J Cell Mol Med. 2022 Apr 1;26(9):2607–19. doi: 10.1111/jcmm.17267 (PMC9077305; doi:10.1111/jcmm.17267)
Supplement: Supplementary file 2 — Supplementary Material [file JCMM-26-2607-s002.pdf]

动物试验伦理审查表

编号(№)：N2020213

申请人填写的相关信息	申请单位：河北以岭医药研究院有限公司新药评价中心	
	申请人学历：研究生　　技术职称：工程师	
	试验名称：杠柳次苷对主动脉缩窄致小鼠慢性心衰的作用	
	试验目的：评价杠柳次苷对主动脉缩窄致小鼠慢性心衰的作用。	
	拟进动物情况　动物来源：北京维通利华实验动物技术有限公司。	
	品种品系：C57BL/6 小鼠　等级：SPF　体重：22-25g	
	数量：140 只（♀0 只；♂140 只）	申请日期：2020 年 12 月 08 日
	进驻日期：2020 年 12 月 ~~08~~ 9 *1 日	结束日期：2021 年 01 月 31 日
	试验要点，包括试验方法、观测指标、试验结束后处死动物的方法等：动物购买后进行检疫和适应性饲养，用异氟烷麻醉后，进行主动脉缩窄（假手术组只开胸，不进行缩窄），造模后 1 周开始灌胃，给药 3 周。末次给药结束后，用异氟烷麻醉，B 超检测后，取血、心脏，将心脏送病理检测。	
	申请人签名：刘媛	
审查依据	1. 该项目是否必须用实验动物进行试验，即能否用计算机模拟、细胞培养等非生命方法替代动物或用低等动物替代高等动物进行试验？<br>2. 表中所填申请人资格和所用动物的品种品系、质量等级、规格是否合适，能否通过改良设计方案或用高质量的动物来减少所用动物的数量？<br>3. 能否通过改进试验方法、调整试验观测指标、改良处死动物的方法，来优化试验方案、善待动物？	
动物室负责人意见：	☑情况属实　□ 情况不属实	签名：王[illegible]　日期：2020.12.08
动物试验伦理委员会意见：	☑情况属实　□ 情况不属实	签名：[illegible]　日期：2020.12.08

*2 打印错误

*1 笔误 刘媛 2020.12.08

*2 笔误 刘媛 2020.12.08

实验动物质量合格证明

专题号：预试	供试品：杠柳次苷
试验名称：杠柳次苷对主动脉缩窄致小鼠慢性心衰的作用	

北京市 实验动物质量合格证

No.110011200109917278

购买单位：河北以岭医药研究院有限公司新药评价中心　　动物实验单位：河北以岭医药研究院有限公司新药评价中心

动物品种品系	等级	动物规格			数量
		体重	日龄	性别	
小鼠, C57BL/6N	SPF级	22-25g		雄性	140
最近一次质量检测日期	2020-10-09	质量检测单位		北京维通利华实验动物技术有限公司	
用途	科学研究	实验单位使用许可证编号			
出售单位（盖章）	北京维通利华实验动物技术有限公司	许可证号		SCXK（京）2016-0006	

北京维通利华实验动物技术有限公司 销售专用章

质量负责人：庄道强　　经手人：王梦玺　　开单日期：2020年12月09日

专题负责人签字：刘[illegible]　　日期：2020年12月09日
