# Supplementary material for: Periplocymarin alleviates pathological cardiac hypertrophy via inhibiting the JAK2/STAT3 signalling pathway
Source: J Cell Mol Med. 2022 Apr 1;26(9):2607–19. doi: 10.1111/jcmm.17267 (PMC9077305; doi:10.1111/jcmm.17267)

Cell Line Authentication Service

STR Profiling Report

Sample From: Shanghai GuanDao Biological Engineering Co., Ltd

Sample Type: Cell Line

Testing Method: STR Genotyping

Report Time: April 23，2021

Cell Line Authentication – STR Profiling Report

Sample code

Table 1. Sample Code

Customer's code	Company Code
H9c2(2-1)	20210423-12

Sample Number:1

Sample Type: Cell line

Testing Type: STR

Testing Method:

Rat STR v2.0 was used to multiplex amplify 9 Rat STR loci (18-3, 9-2, 6-7, 5-5, X-1, 15-3, 12-1, 6-4, 4-2), 2 human STR loci (CSF1PO, vWA) and sex locus Jarid1. The PCR products were detected by ABI 3730xl genetic analyzer, and the detection results were analyzed by GeneMapperID-X software (Applied Biosystems).

Test Results

1. STR profile

Table 2. STR and Amelogenin Genotyping Results of Cell line 20210423-12.

Loci	Sample information			Cell Bank information		
	Sample name：H9c2(2-1)			Cell line name：		
	**Allele1**	**Allele2**	**Allele3**	**Allele1**	**Allele2**	**Allele3**
Rat STR 18-3	17	17				
Rat STR 9-2	12.3	12.3				
Rat STR 6-7	13	13				
Rat STR 5-5	15	15				
Rat STR X-1	23.3	25.3				
Rat STR 15-3	22.3	24.3				
Rat STR 12-1	14.3	16.3				
Rat STR 6-4	16.3	19.3				
CSF1PO						
VWA						
Rat STR 4-2	17.3	17.3				
Rat STR Jarid1	X	X				

2. Authentication

☑ The submitted sample profile is Rat, but not a match for any profile in the Cellosaurus. As the STR database of **H9c2(2-1)** was not logged in, the matching result can not be displayed. No cross contamination was found in the cell line , if the paper was to publish, these data could be submitted to the magazine.

☐ The submitted profile is exact match for the following Rat cell line(s) in the Cellosaurus STR database (8 core loci plus Amelogenin): /**.**

☐ The submitted profile is similar to the following Cellosaurus Rat cell line: /.

- **Note:** A cell line can be considered to be authenticated when 80% (exact match) of the alleles in its STR profile match profiles from tissue or other cell line samples from that donor or from database. Cell lines with between a 55% to 80% (similar) match require further profiling for investigation of relatedness.

Prompt Description:
1.The results of negative and positive controls in the experiment are correct;
After DNA amplification of the detected sample cells, the map is clear and the typing result is good;
2.The Rat-derived site of the cell showed a peak, but the human-derived site did not show a peak;
3.There are 0 multiple alleles in 9 Rat-derived loci in this cell;
4.The STR locus typing results of this cell are shown in the attached table, and the typing map is shown in the attached figure;

The STR typing results of rat cells with the DNA of this cell showed:1.The cells are rat cells;2.No human cell contamination was found in this cell;3.No cross-contamination of rat cells was found in the cells

Comments:
1.Single-source rat cells: 9 loci and sex loci from mice showed single or double peaks, while human loci did not show peaks;
2.Identification of human pollution: if all 12 sites have peaks, it means that the detected cells are a mixture of human and rat cells; If only 2 human origin sites and sex sites have peaks, it means that the detected cells may be human origin cells;
3.Identification of contamination in rats: More than or equal to 3 multi-alleles in 9 specific loci in rats suggest that there may be cross-contamination of homologous species, and 1-2 multi-alleles may originate from cell variation and are not regarded as contamination;
4.The effective peaks are true PCR bands, and small peaks and nonspecific bands are ignored in the calculation;

Appendix:

1. Genotyping Strategy and Site Distribution

Table S1. Experimental Strategy and Sites

	Strategy 1	Strategy 2	Strategy 3
1	18-3	15-3	VWA
2	9-2	12-1	4-2
3	6-7	6-4	Jarid1
4	5-5	CSF1PO	
5	X-1		
6			

The allele match algorithm compares the 12 core loci plus amelogenin only, even though alleles from all lociwill be reported when available.

2. Cellosaurus tools was used to carry on the cell line comparison, which contains cell lines STR data from ATCC, DSMZ, JCRB ,ECACC， GNE and RIKEN databases. If the cell is not included in the above cell library, users need to compared with other databases.

Technician: Xiaolu Li

Checked by: Liqun Zhao

Issued by: Xue Han

Issue date: April 23， 2021

Applied Biosystems
GeneMapper® ID-X 1.2

Sample Name	SQO	SOS	SQ	SSPK	MIX	OMR	CGQ
12							

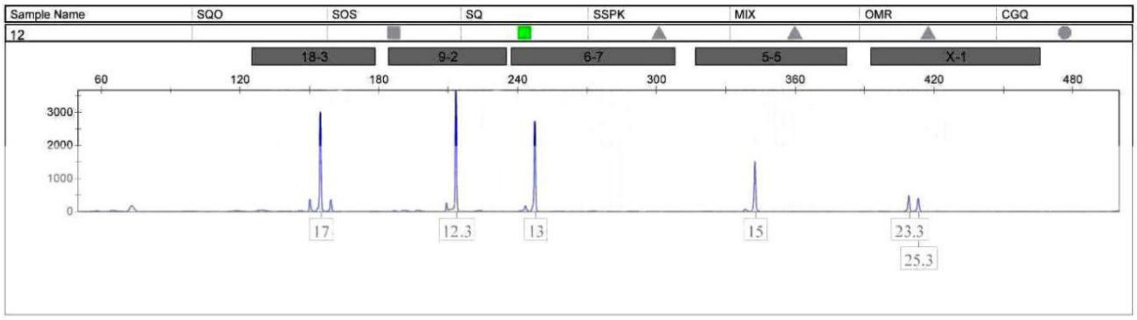

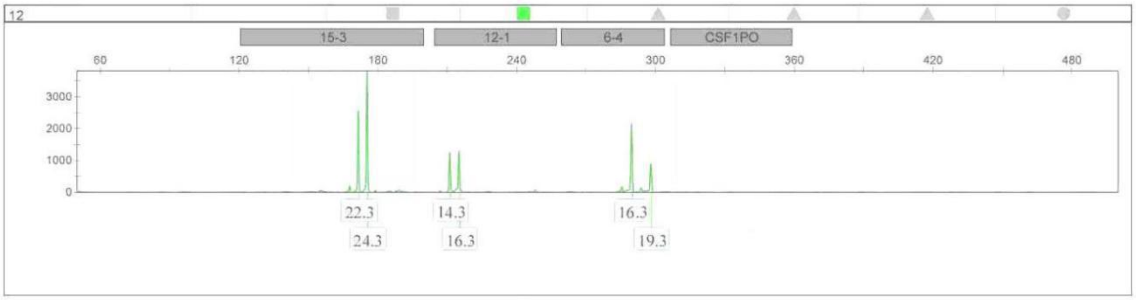

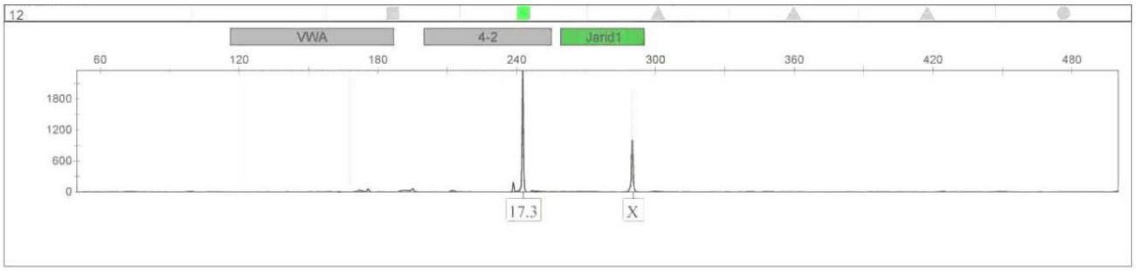

Supplement: Supplementary file 3 — Supplementary Material [file JCMM-26-2607-s003.pdf]
